# Gene Electrotransfer via Conductivity‐Clamped Electric Field Focusing Pivots Sensori‐Motor DNA Therapeutics: “A Spoonful of Sugar Helps the Medicine Go Down”

**DOI:** 10.1002/advs.202401392

**Published:** 2024-06-14

**Authors:** Jeremy L. Pinyon, Georg von Jonquieres, Edward N. Crawford, Amr Al Abed, John M. Power, Matthias Klugmann, Cherylea J. Browne, David M. Housley, Andrew K. Wise, James B. Fallon, Robert K. Shepherd, John Y. Lin, Catherine McMahon, David McAlpine, Catherine S. Birman, Waikong Lai, Ya Lang Enke, Paul M. Carter, James F. Patrick, Robert D. Gay, Corinne Marie, Daniel Scherman, Nigel H. Lovell, Gary D. Housley

**Affiliations:** ^1^ Translational Neuroscience Facility Department of Physiology School of Biomedical Sciences Graduate School of Biomedical Engineering Tyree Institute for Health Engineering (IHealthE) UNSW Sydney NSW 2052 Australia; ^2^ Charles Perkins Centre School of Medical Sciences Faculty of Medicine and Health University of Sydney Sydney NSW 2006 Australia; ^3^ Medical Sciences School of Science Western Sydney University Sydney NSW 2560 Australia; ^4^ Bionics Institute 384–388 Albert Street East Melbourne VIC 3002 Australia; ^5^ Medical Bionics Department of Otolaryngology University of Melbourne Melbourne VIC 3002 Australia; ^6^ Tasmanian School of Medicine University of Tasmania Hobart TAS 7001 Australia; ^7^ Faculty of Medicine and Health Sciences The Hearing Hub Macquarie University Sydney 2109 Australia; ^8^ Faculty of Medicine and Health University of Sydney Sydney NSW 2006 Australia; ^9^ Department of Otolaryngology Royal Prince Alfred Hospital Camperdown NSW 2050 Australia; ^10^ NextSense Royal Institute of Deaf and Blind Children Gladesville NSW 2111 Australia; ^11^ Cochlear Limited Macquarie University University Avenue Macquarie Park NSW 2109 Australia; ^12^ CNRS, Inserm, UTCBS Université Paris Cité Paris F‐75006 France; ^13^ Chimie ParisTech Université PSL Paris 75005 France; ^14^ Fondation Maladies Rares 96 rue Didot Paris 75014 France

**Keywords:** CNS electrotransfer, CNS neuromodulation, nerve regeneration, nonviral gene augmentation therapy, precision gene delivery

## Abstract

Viral vectors and lipofection‐based gene therapies have dispersion‐dependent transduction/transfection profiles that thwart precise targeting. The study describes the development of focused close‐field gene electrotransfer (GET) technology, refining spatial control of gene expression. Integration of fluidics for precise delivery of “naked” plasmid deoxyribonucleic acid (DNA) in sucrose carrier within the focused electric field enables negative biasing of near‐field conductivity (“conductivity‐clamping”–CC), increasing the efficiency of plasma membrane molecular translocation. This enables titratable gene delivery with unprecedently low charge transfer. The clinic‐ready bionics‐derived CC‐GET device achieved neurotrophin‐encoding miniplasmid DNA delivery to the cochlea to promote auditory nerve regeneration; validated in deafened guinea pig and cat models, leading to improved central auditory tuning with bionics‐based hearing. The performance of CC‐GET is evaluated in the brain, an organ problematic for pulsed electric field‐based plasmid DNA delivery, due to high required currents causing Joule‐heating and damaging electroporation. Here CC‐GET enables safe precision targeting of gene expression. In the guinea pig, reporter expression is enabled in physiologically critical brainstem regions, and in the striatum (globus pallidus region) delivery of a red‐shifted channelrhodopsin and a genetically‐encoded Ca^2+^ sensor, achieved photoactivated neuromodulation relevant to the treatment of Parkinson's Disease and other focal brain disorders.

## Graphical abstract

Precision naked deoxyribonucleic acid (DNA) DNA gene delivery is achieved through the development of “conductivity‐clamped gene electrotransfer (CC‐GET)” utilizing a bionic array electro‐lens device that biases local conductivity to the polyanionic therapeutic nucleic acid molecules, enhancing pulsed electric field strength with minimal charge transfer. CC‐GET is applied to neurotrophin‐mediated auditory nerve repair to improve bionic hearing and surmounts the biophysical barriers to targeted delivery of naked DNA gene constructs to the brain.

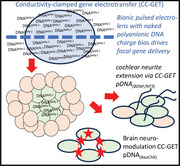

## 1 Introduction

Delivery technologies are central to enabling deoxyribonucleic acid (DNA) and ribonucleic acid (RNA)‐based therapeutics. “Pre‐Covid,” viral vectors were the most widely adopted vehicle,^[^ ^1^ ^]^ while nanoparticle packaging now dominates RNA therapeutics and is increasingly being utilized in clinical trials.^[^ ^2^ ^]^ These technologies arevariously challenged by uncontrolled dispersion of the active agent, packaging limitations, stability, liver toxicity, immuno‐incompatibility (affecting present and future treatments), and cost.^[^ ^3^ ^]^ Gene electrotransfer (GET) offers an alternative delivery strategy, where electric pulses cause naked polyanionic nucleic acid molecules (NAs) to bind to the cathode‐facing plasma membranes of cells in target tissues.^[^ ^4^ ^]^ This delivery is virtually instantaneous and modulated by electric field strength. The membrane‐bound NAs undergo endocytosis, and in the case of DNA, a fraction is transported to the nucleus to support episomal gene transcription.^[^ ^5^ ^]^ GET is distinct from transient pore formation in the plasma membrane, termed “electroporation,” which is generated by high voltage pulsed electric fields and enables translocation of low molecular weight molecules.^[^ ^6^, ^7^ ^]^ This is exemplified by the demonstration of progressive take‐up of the impermeant fluorescent dye propidium iodide following delivery of the pulsed electric field (via transient and irreversible electroporation), while (fluorescently tagged) naked DNA binding to the plasma membrane is limited to the duration of the pulse train,^[^ ^6^ ^]^ followed by slower internalization in association with clathrin and caveolin‐/lipid raft – mediated endocytosis; validated by dose‐dependent pharmacological inhibition of reporter gene expression.^[^ ^5^ ^]^

At present, clinical applications for GET are primarily focused on oncology, notably supporting the delivery of DNA vaccines driving immunotherapies, such as interleukin 12‐encoding DNA to stimulate innate immune responses to melanoma using devices with multiple needle electrodes for “open‐field” electrotransfer.^[^ ^8^ ^]^ There are a range of medical devices being used in clinical trials for reversible and irreversible electroporation, GET, and electrochemotherapy applications.^[^ ^3^, ^9^ ^]^ The invasiveness of the electrode configurations for “open‐field” GET, and the requirement for high voltages and currents to generate efficacious pulsed electric fields, cause tissue injury,^[^ ^10^ ^]^ limiting the application scope, and are painful. However, “close‐field” GET has been established by electric field focusing (local electric field compression) utilizing multi‐channel linear electrode arrays (derived from cochlear implant technology), where the target tissue surrounding the GET probe is exposed to an orthogonal‐compressed electric field, rather than the homogenous electric field exposure inherent to having the target tissue between two or more electrodes.^[^ ^11^, ^12^, ^13^, ^14^, ^15^ ^]^

Here we describe the enablement of enhanced close‐field GET which incorporates clamping of conductivity within the focused electric field, using a sucrose carrier for the negatively charged plasmid DNA. This enhances the electric field focusing and minimizes charge transfer to achieve suprathreshold electric field strength. The efficacy and safety of this conductivity‐clamped gene electrotransfer (CC‐GET) technology were evaluated in guinea pig and cat cochlea models supporting the development of a clinical CC‐GET gene delivery device and neurotrophin‐encoding therapeutic miniplasmid DNA, for application in regenerative medicine to regrow the atrophied auditory nerve in patients receiving a cochlear implant (CI), closing the “neural gap” to enhance the “bionic ear” neural interface. The utility of CC‐GET was further validated in the brain, a tissue particularly vulnerable to the high voltage/current profiles utilized for conventional GET. Studies here demonstrate that CC‐GET in the guinea pig brain enables the safe targeted expression of reporter and optogenetic gene cassettes at charge transfer levels which would be ineffective using conventional GET. The development of CC‐GET establishes a pathway for complementary gene therapy to augment cochlear and brain bionics and more broadly to achieve spatially controlled titratable DNA electrotransfer for sensorimotor disorders, and other deep‐tissue pathophysiological targets throughout the body.

## 2 Results and Discussion

### 2.1 Localized Conductivity‐Clamping Enhances Array‐Based Electric Field Compression for DNA Electrotransfer

**Figure 1:**
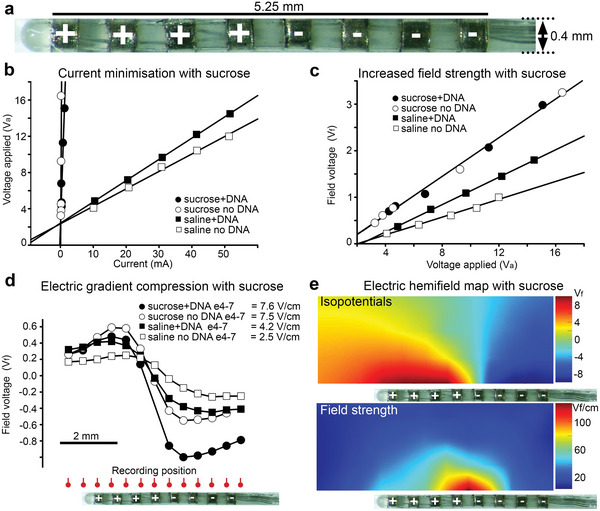
Nonconductive sucrose carrier amplifies bionic array electric field strength. a) Image of an 8‐electrode GET array (derived from a preclinical cochlear implant). b) Constant current pulses of increasing amplitude across the ganged electrodes in normal saline, or the nonconducting 10% sucrose carrier, with or without DNA (2 µg µL^−1^) generate applied voltages (*V*_a_) on the electrodes. Note the amplification of *V*_a_ provided by “conductivity‐clamping” using a sucrose carrier. c) Sucrose‐enhanced field voltage (*V*_f_) measured at a reference point 0.5 mm lateral to electrode 7 (second from left in (A)), compared with saline carrier. d) V_f_ sampled along the array demonstrates the electric “lensing” effect, with the greatest change in voltage/slope around the null point in the middle of the array. DNA + sucrose ∼ doubled the electric field strength (∆*V*_f_/*∆*d) between electrodes 4–7 compared with saline + DNA. e) Isopotential map (hemifield) and derived electric field map for sucrose carrier (40 V applied, 100 ms pulses). Data from B to E are reflected in the US patent.^[^ ^16^ ^]^ Data for 0.9% NaCl without DNA in (D) is ref. [14]. See also Supporting Information primary figure commentary.

The CC‐GET device utilizes a linear electrode array to create an “electro‐lens” that compresses the electric field orthogonally, for optimal plasmid DNA electrotransfer efficiency, where the electric field strength (*V*_f_/cm) is the driver for the molecular translocation of DNA to the cell.^[^ ^12^ ^]^ Complementing this electric field shaping, a linear electrode array lends itself to the use of low‐conductance DNA carrier solution in the immediate proximity to the electrodes (“conductivity‐clamping”) to enhance the electric field strength and hence electrophoretic delivery of DNA to the plasma membrane. The clamping of extracellular conductivity using isotonic sucrose carrier with negatively charged DNA as the major charge species was evaluated to determine the effect on the voltage in close‐field (**Figure** **1**).

The relationship between constant current pulse amplitude and the applied voltage (*V*_a_) at the GET array (Figure 1a) (delivered by an analog‐controlled Digitimer DS5 stimulator in voltage mode) was compared between use of 2 µg µL^−1^ DNA (salmon sperm sheared DNA) in isotonic sucrose (10%) and normal saline (0.9% NaCl) + DNA (Figure 1b). The sucrose carrier increased resistivity across the array by ≈43‐fold (sucrose + DNA = 12.08 kΩ; 0.9% saline + DNA 0.28 kΩ). Thus, by way of example, ≈14.5 V applied voltage (*V*_a_) would elicit a 1.2 mA current pulse in sucrose + DNA, whereas this *V*_a_ would cause a 51.6 mA current pulse in saline + DNA. By comparison, in the absence of DNA, the array resistance in sucrose was 78.38 kΩ, compared with 0.23 kΩ for saline only; thus DNA was the principal charge species in sucrose (650% increase in conductivity), compared with saline (20% change). Further, the sucrose carrier provided an added advantage with respect to electric field strength, where the voltage in the field (*V*_f_) measured adjacent to the electrode array was 42% greater compared with using saline at equivalent *V*_a_ (Figure 1c). Measured 0.5 mm orthogonal to electrode 7 (anode # 3), sucrose + DNA = 0.209 *V*_f_/*V*_a_ compared to 0.147 *V*_f_/*V*_a_ for saline + DNA; reflecting the differential conductivity. In summary, the substitution of sucrose + DNA as a carrier over conventional saline + DNA provides ≈75× increased benefit in effective voltage achieved in the field for equivalent current delivery. Given that joule heating of tissue is dependent on current squared multiplied by resistance, minimization of current using sucrose carrier represents a significant advantage.

Extending from these reference point measurements, the change in *V*_f_ adjacent to all eight electrodes in the array was measured using 100 ms × 5 V pulses; sampling at 0.5 mm steps, 0.5 mm lateral to the array (Figure 1d). This demonstrated that adding DNA to the saline carrier increased the maximum electric field strength (slope) by 68% (4.2 V cm^−1^ compared with 2.5 V cm^−1^, as peak slope about the null point between the four ganged anodes and four ganged cathodes, sampled at ≈95 ms). In comparison, the use of sucrose carrier + DNA at the same applied voltage (5 V), further increased the field strength (7.6 V cm^−1^) by 81% above the saline + DNA level. Electric field mapping was then undertaken adjacent to the electrode array using a translational stage at 0.5 mm resolution steps, with 10% sucrose carrier, using 100 ms × 40 V pulses, which is the suprathreshold for DNA electrotransfer^[^ ^12^ ^]^ (Figure 1e). This demonstrated the spherical electric field lensing effect of the DNA electrotransfer array in the sucrose carrier.

### 2.2 Validation of High Efficiency Gene Expression Using CC‐GET

**Figure 2:**
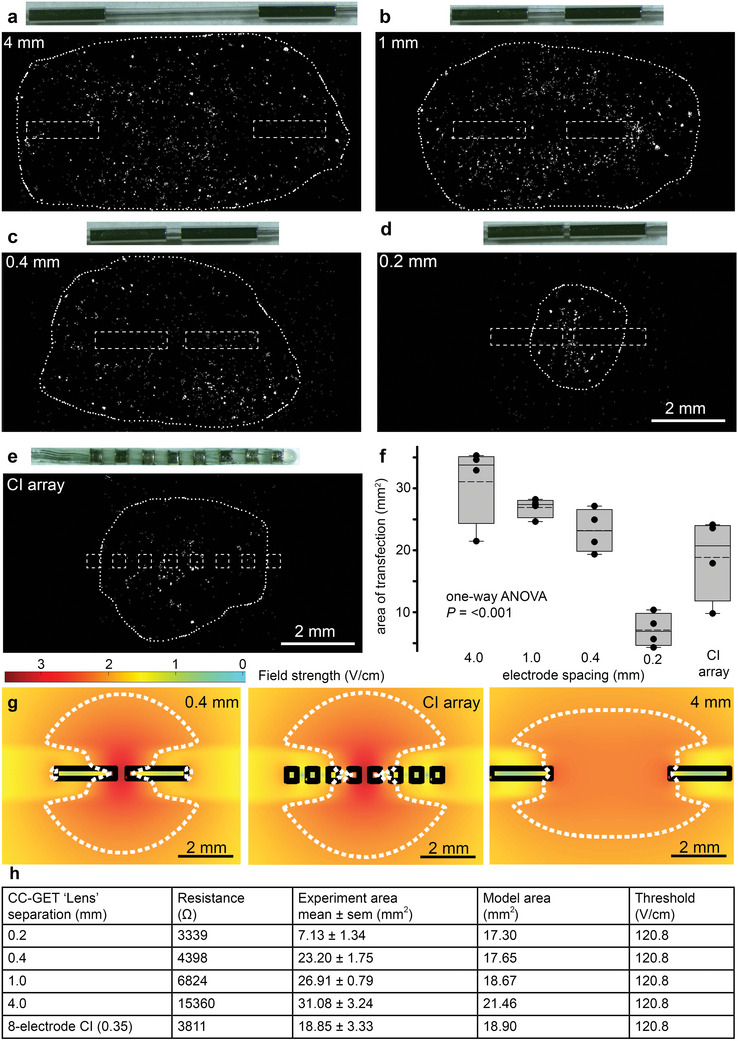
Electric field “lensing” via the CC‐GET array enables spatial focusing of gene expression. a–d) Variation in the separation between the extended conductive surfaces of the linear CC‐GET array (modeling the ganged discrete electrode elements as shown in (e), acts as a “lens” to change the spread of the electric field for the same charge transfer, where the CMVp‐hrGFPnls plasmid DNA in 9% sucrose (0.09% NaCl) was delivered to HEK293 cell monolayers (10 × 100 µs, 400 µs interpulse interval, 30 mA square wave pulses; requiring 120 V applied; ≈30 µC total charge transfer; ≈4 kΩ local resistance). f) Boxplots of areas of transfected cells relative to electrode separation show 25% and 75% boundaries, with 95% limits as bars; lines indicate median, dashed lines indicate means, data overlaid (*n* = 4 per group). g) Monte Carlo modeling of electric fields across the configurations. The dashed perimeter, established by comparison with the perimeter of the fields of the bioreporter transfected HEK293 cells reflects the electric field threshold for DNA electrotransfer (120.8 V cm^−1^). h) The predicted electric field threshold was validated in silico by further simulations using the clinical CC‐GET device. See also Supporting Information – methods and primary figure commentary.

Given the evident improvement in electric field strength achieved by local clamping of conductivity using a 10% sucrose carrier, CC‐GET was functionally validated in a human embryonic kidney 293 (HEK293) cell monolayer model (after ref. [12]). These experiments showed that sustained exposure of these cells to 10% sucrose + CMVp‐hrGFPnls plasmid DNA (2 µg µL^−1^; Figure S1A, Supporting Information) was innocuous and demonstrated significantly improved gene expression over the use of a conventional saline‐based carrier. This was demonstrated though the increased surface area of the circular field of green fluorescent protein (GFP)+ve transfected cells centered on the null point of the DNA delivery probe between the ganged anodes and cathodes (sucrose carrier = 21.7 ± 2.8 mm^2^ GFP+ve cells, *n* = 5; saline = 13.5 ± 1.3 mm^2^ GFP+ve cells, *n* = 9; *p* = 0.0103, Student's *t*‐test; 3 × 100 ms × 35 V pulses; Figure S2A, Supporting Information). This represented a 27% expansion in the diameter of the field of transfected HEK293 cells for the same fixed *V*_a_; where the perimeter reflects the boundary electric field threshold for gene electrotransfer (e.g., ≈2.5 mm distal to the array centroid in the case of 10% sucrose carrier). This GFP reporter study, with 3 days in cell culture, validated the field‐strength gain attributable to local conductivity‐clamping, at a fixed voltage, determined in the biophysical measurements shown in Figure 1c,d. This model was then extended to quantify the efficiency of GET within the field of GFP‐positive cells using the same pulse parameters and reporter plasmid, comparing saline versus sucrose carriers. The cells were fixed after 24 h (to limit cell division^[^ ^17^ ^]^) and the nuclei were labeled with DAPI (4'‐6'‐diamino‐2‐phenylindole). A prescribed region of interest (ROI) within the field of GFP‐expressing cells was imaged to provide counts for GFP‐positive cells and GFP‐negative – DAPI‐only cells. These data (Figure S2A–C, Supporting Information) replicated the original finding of increased area of GFP cell expression with sucrose carrier (sucrose = 14.25 ± 0.98 mm^2^; saline = 6.97 ± 1.17 mm^2^; *n* = 6 per group; *p* = 0.000733, *t*‐test). Two‐way ANOVA of the cell count data for GFP positive cells and DAPI positive (GFP negative) cells showed a significant impact of GET on cell survival using a saline carrier (sucrose GFP = 141.83 ± 34.90 cells; saline GFP = 73.83 ± 11.43 cells; sucrose DAPI = 538.00 ± 112.18 cells; saline DAPI = 286.67 ± 57.66 cells; *n* = 6 per group; *p* = 0.0175 Holm‐Sidak post hoc comparison), (Figure S2B–D, Supporting Information). While cell density was reduced with saline‐based GET, relative gene expression efficiency within the ROIs (% GFP positive cells / total cell number) was equivalent for both sucrose and saline carriers (sucrose = 20.349 ± 1.056% efficiency; saline = 21.792 ± 2.812% efficiency; *p* = 0.641, *t*‐test; Figure 2d).

The evident impact of saline carrier on cell density was investigated using an experimental design where, 1 h after GET using either sucrose or saline carriers, the cell impermeant fluorescent marker propidium iodide (PI) was added to the culture media for 5 min, to label cells with irreversible permeabilization, followed by fixation and imaging. Conductivity – clamping provided by the sucrose carrier was determined from local resistance measurements, showing a 92% average reduction in current for the 35 V pulses (sucrose = 2.76 ± 0.03 mA; saline = 35.58 ± 1.98 mA; *p* < 0.001 *t*‐test). This study demonstrated a significant injury margin for the saline carrier, proximal to where the GET electrode array had been positioned over the cell monolayer; consistent with known irreversible electroporation arising from high current flux.^[^ ^18^ ^]^ The PI signal was minimal for the sucrose carrier, where the measured margin largely represented mechanical injury from electrode placement. Average PI areas: saline = 10.23 ± 1.62 mm^2^ (*n* = 6); sucrose = 2.58 ± 0.47 mm^2^ (*n* = 5); *p* = 0.00245 *t*‐test, (Figure S2E–G, Supporting Information).

Having identified the advantage of CC‐GET, we sought to establish the minimum (and hence safest) charge delivery protocol for effective gene expression. Experiments were performed using CMVp‐hrGFPnls plasmid (2 µg µL^−1^) with HEK293 cell monolayers, varying current amplitude applied to the clinical CC‐GET DNA delivery probe with 3 × 100 µs square wave pulses (Figure S3, Supporting Information). This demonstrated a threshold of ≈5 mA (Figure S3B, Supporting Information) (*p* = 0.00017, single sample *t*‐test), with a field of GFP+ve cells of 0.77 ± 0.08 mm^2^ (*n* = 6), with a nonlinear increase to 12.3 ± 1.9 mm^2^ (*n* = 5) using 15 mA pulses (*p* = 0.002, one way ANOVA on ranks) (Figure S3D, Supporting Information).

The scalability of the electric field created by the linear DNA electrotransfer array was determined by simplification of the array from a multi‐node “tandem” electrode configuration to a device that comprised two discrete elongated current donors and receiver Pt/Ir electrode surfaces (2 mm length, 400 µm diameter), where systematic changes in the dispersion of the electric field “lens” was achieved by separation of these two electrodes on a silicone core backbone. These experiments showed that the “lensing” for focused electric field GET was controllable by adjusting the separation between the electrode poles (**Figure** **2**a–h). For a fixed charge transfer, the surface area of transduced HEK293 cells expressing the GFP reporter varied around fourfold, from (7.13 ± 1.34 to 31.08  ± 3.24 mm^2^), with lens separation of 0.2–4.0 mm respectively; representing a decompression of the electric field (Figure 2g). In conjunction with the Monte Carlo simulation of the electric fields (Figure 2h), the perimeters of GFP+ve transfected HEK293 cells established the electric field boundary condition for DNA electrotransfer at *V*_f_/d = 120.8 ± 20.2 V cm^−1^ (mean ± SD). These experiments utilized 30 mA constant current (100 µs) delivered in a 9% sucrose + 0.09% NaCl solution (modeling physiologically relevant conductivity‐clamping) via 120 V applied driving voltage. These findings highlight the capability of CC‐GET to achieve “dial‐up” spatial control of gene expression with minimal charge transfer.

The scala tympani fluid compartment of the human cochlea that receives the CI varies in diameter from ≈2.5 mm at the base to ≈0.75 mm at the upper insertion position for a CI array (25 mm in length).^[^ ^19^ ^]^ These experiments confirmed that the ganged four‐electrode configuration for the anode and cathode elements of the GET array, with a 350 µm separation between the anode and cathode gangs, as previously used in initial proof of concept studies,^[^ ^11^, ^12^, ^14^, ^15^, ^17^ ^]^ provided an ideal electric field shape to achieve the targeted delivery of naked DNA encoding neurotrophins to the scala tympani mesenchymal cells immediately adjacent to a subsequently inserted (permanent) CI array. The bionic arrays used for chronic deep brain stimulation (DBS) to manage Parkinson's Disease dyskinesia utilize a similar electrode array configuration and pulse parameters^[^ ^20^ ^]^ and hence the tuning of the CC‐GET array was predicted to enable safe and effective DNA delivery to the brain.

### 2.3 Development of a Clinical CC‐GET Medical Device for Auditory Nerve Regeneration to Augment Bionic Ear Functionality

**Figure 3:**
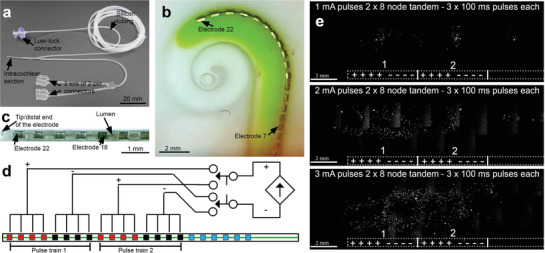
Design of the clinical CC‐GET linear array for scalable targeting of plasmid DNA delivery to the cochlea. a) Intracochlear array of 22 half‐band Pt/Ir electrodes incorporates a lumen that delivers the DNA in sucrose carrier solution via the tip (provided by Cochlear Ltd.). b) Image from a modeled human cochlea scala tympani fluid compartment, showing insertion of the CC‐GET array, with green dye demonstrating how, in clinical application, the DNA in sucrose carrier solution would displace the perilymph and clamp the conductivity around the array, resulting in amplification of the electric field produced by the constant current pulses. c) Detail of the tip of the clinical CC‐GET array showing the electrode elements and the internal lumen. d) Diagram of array “zone” switching to enable successive DNA electrotransfer to adjacent zones (e22–e15; e14–e7). e) CMVp‐hrGFPnls plasmid DNA electrotransfer to HEK293 cell monolayers with increasing current. Data from (E) are reflected in US patent ref. [16]. Modeling of the electric field generated for GET by this device within the guinea pig cochlea is described in ref. [13].

Development of the clinical CC‐GET device was achieved by biomedical engineers at Cochlear Ltd by reengineering a conventional 22 half‐band Pt/Ir CI array (Cochlear Nucleus CI622 array derivative; Cochlear Ltd) to produce the 2 × 4 ganged electrodes at 0.4 mm “lens” spacing as above (electrodes e15–e22), with the addition of a second pair of ganged electrodes (e7–e14). This arrangement enables more basal targeting of DNA electrotransfer by switching between array elements and delivering a second pulse train (**Figure** **3**). To achieve localized conductivity‐clamping, the microfluidic delivery capability was integrated into the CC‐GET array by molding a lumen into the silicone, beneath the half‐banded electrodes, enabling local displacement of the cochlear perilymph solution with the therapeutic DNA in 10% sucrose carrier. Given that the scala tympani compartment is effectively blind‐ended, DNA delivery from the tip of the inserted array results in DNA solution displacing cochlear perilymph around the CC‐GET device (Figure 3b), clamping the electric field as modeled. The efficacy of the switching between successive electrode elements within the cochlear CC‐GET device to achieve DNA electrotransfer along the length of the perilymphatic compartment was modeled in vitro (Figure 3e). Nuclear localized GFP fluorescence of HEK293 cells showed titratable “painting” of DNA electrotransfer over an extending distance when a pulse train (3 × 100 ms) was delivered to the apical‐most ganged electrode set (e15–e22) and then the more basal set (e7–e14) (1–3 mA range), modeling electrotransfer to the mesenchymal cells of the cochlea scala tympani immediately adjacent to the CC‐GET device.

### 2.4 CC‐GET Targeting Guinea Pig Cochlear Perilymphatic Mesenchymal Cells In Vivo

A 4‐month in vivo safety and efficacy study was conducted to validate CC‐GET for targeted gene expression in the mesenchymal cells of the guinea pig cochlea scala tympani compartment. These cells are the target for neurotrophin gene augmentation, as they are closest to the CI array, and their secretion of the recombinant neurotrophins establishes a gradient for directed regeneration of the atrophied spiral ganglion afferent fibers, closing the neural gap with the CI array (after ref. [11]). These experiments were undertaken in normal hearing guinea pigs using an enhanced GFP (eGFP) reporter sequence incorporated into the pFAR4 miniplasmid backbone (**p**lasmid **F**ree of **A**ntibiotic **R**esistance; after ref. [21]). The pFAR4 plasmid is compliant with the European Medicines Agency guidelines limiting the use of plasmids which could result in misdirected electrotransfer of antibiotic resistance genes to commensal bacteria.^[^ ^22^ ^]^ The experiments used the clinical CC‐GET device with an optimized pulse train (10 × 100 µs capped at 50 mA). The custom control system developed for the clinical application captured the current and voltage parameters for each current pulse delivered, which enabled measurement of the local resistance via the CC‐GET array. The geometry of the guinea pig cochlea constrained the electrotransfer to the basal turn region, which was targeted using the apical eight‐ganged electrodes of the clinical CC‐GET device (zone 1 of Figure 3d). The DNA electrotransfer of the pFAR4‐CMVp‐eGFP miniplasmid (Figure S1B, Supporting Information) was performed under isoflurane anesthesia by insertion of the CC‐GET array into the cochlear scala tympani via a perforation in the round window membrane. Fifty microliters of the DNA solution (2 µg µL^−1^ pFAR4‐CMVp‐eGFP DNA in 10% sucrose; 0.5 mm NaOH (added to achieve physiological pH)) was delivered at 10 µL min^−1^. The displacement of cochlear perilymph and enhancement of the electrotransfer biophysical profile achieved by conductivity‐clamping via sucrose carrier infusion into scala tympani was confirmed by comparison of resistance just prior to commencing DNA infusion (mean = 770 ± 60 Ω) with the increased resistance at the time of electrotransfer (mean = 3.78 ± 0.56 kΩ; *n* = 3; *p* = 0.0391, paired *t*‐test, *n* = 3; Table S1, Supporting Information). Across 12 experiments, the applied voltage required to achieve the constant current pulses ranged from 70 to 120 V (*V*_a_ mean = 103.4 ± 5.51 V), where the upper limit reflected the compliance limit of the constant current stimulator (120 V; current pulse range 29–50 mA; mean = 45.1 ± 1.98 mA) dictated by the local resistance achieved by infusion of the sucrose carrier (2.40 ± 0.24 kΩ) (Table S1, Supporting Information). The resulting expression of the eGFP reporter was determined by hemi‐sectioning decalcified cochleae and acquiring confocal images that captured the mesenchymal cell surface lining the perilymphatic scalae. Figure S4 (Supporting Information) shows the pronounced eGFP reporter fluorescence 4 days post CC‐GET, with a significant drop‐off by 2 weeks, and minimal cells expressing GFP at 16 weeks (*p *= 0.031, ranked ANOVA). These data are consistent with the turnover of the cochlear mesenchymal cells as previously described.^[^ ^17^ ^]^ Here, using CC‐GET, reliable GFP expression was achieved using 45 µC charge transfer, which is close to a thousand‐fold improvement over the ≈40 mC minimum effective charge delivery previously required for close‐field GET to cochlear mesenchymal cells without conductivity‐clamping.^[^ ^11^ ^]^

### 2.5 Auditory Nerve Regeneration in the Deafened Guinea Pig Cochlea Using a Therapeutic Humanized Dual Neurotrophin‐Encoding Miniplasmid DNA

**Figure 4:**
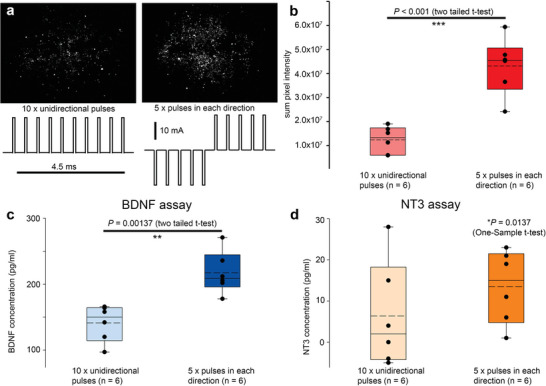
Validation of recombinant BDNF and NT3 protein synthesis using HEK293 cells and the clinical CC‐GET device to deliver pFAR4‐CMVp‐BDNF‐NT3 miniplasmid and pBs‐CMVp‐mCHERRYnls reporter DNA. a,b) Comparison of mCherry reporter expression using positive‐going, versus positive then negative pulses for GET (100 µs × 20 mA; 400 µs interpulse interval). c,d) Validation of BDNF and NT3 secretion using both pulse train profiles. Data reflected in Figure 12 Patent ref. [23]. Cells were imaged and supernatant was collected for ELISA at 4 days post CC‐GET. See also Supporting Information – Methods and Primary Figure Commentary.

The efficacy of our candidate therapeutic neurotrophin miniplasmid DNA (pFAR4‐CMVp‐BDNF‐IRES‐NT3; Figure S1C, Supporting Information) for auditory nerve regeneration was evaluated in the deafened guinea pig model (*n* = 4). This molecule includes a bicistronic expression cassette comprising codon‐optimized human brain‐derived neurotrophic factor (BDNF) and neurotrophin‐3 (NT3) elements. Prior to the in vivo experiments, secretion of the recombinant BDNF and NT3 neurotrophins was validated in HEK293 cells via ELISA (**Figure** **4**). These in vitro experiments included the use of trains of monophasic, or varying polarity, pulses to demonstrate a correlation between the expression of a cocktail of a CMVp‐mCHERRYnls reporter plasmid (Figure S1D, Supporting Information) alongside the clinical neurotrophin miniplasmid pFAR4‐CMVp‐BNDF‐IRES‐NT3. These HEK293 cell‐based assays indicated that BDNF secretion was significantly greater than NT3 secretion, as anticipated from the latter being downstream of the IRES element.

This dual neurotrophin‐encoding plasmid extended the previously validated efficacy of GET driving BDNF expression for rapid auditory nerve regeneration^[^ ^11^ ^]^ with the addition of the NT3 expression element, which is known to have substantial trophic activity in the cochlea acting synergistically with BDNF treatment to enhance auditory nerve regeneration.^[^ ^24^ ^]^ In vivo delivery parameters for pFAR4‐CMVp‐BDNF‐NT3 DNA were matched to the pFAR4‐CMVp‐eGFP study, with equivalent voltages achieved (Table S1, Supporting Information; mean current amplitude for four deafened guinea pigs = 30.8 ± 2.92 mA, to achieve a maximum applied voltage of 120 V for DNA delivery). The readout from these experiments included ELISA quantitation of BDNF and NT3 levels in cochlear perilymph 2 weeks post‐CC‐GET (1 month after deafening), PCR screening for residual recombinant plasmid DNA in tissues (brain, heart, blood, mastoid process muscle, liver, kidney, stool, and urine), which did not detect the transgene DNA in any of the tissue samples, and immunofluorescence imaging of the spiral ganglion in cryosectioned cochlear tissue. In the four deafened guinea pigs the pFAR4‐CMVp‐BDNF‐NT3 DNA CC‐GET – treated cochleae showed ≈3x higher BDNF levels than the corresponding baseline levels in the untreated cochleae (Figure S5, Supporting Information) (control = 565 ± 396 pg mL^−1^ BDNF; neurotrophin DNA treated cochleae = 1674 ± 356 pg mL^−1^ BDNF; *p* = 0.0189, two‐tailed paired *t*‐test). There was no significant difference between NT3 levels across treated and untreated cochleae (control = 162 ± 64 pg mL^−1^ NT3; neurotrophin DNA treated cochleae = 165 ± 43 pg mL^−1^ NT3; *p* = 0.941, two‐tailed paired *t*‐test).

**Figure 5:**
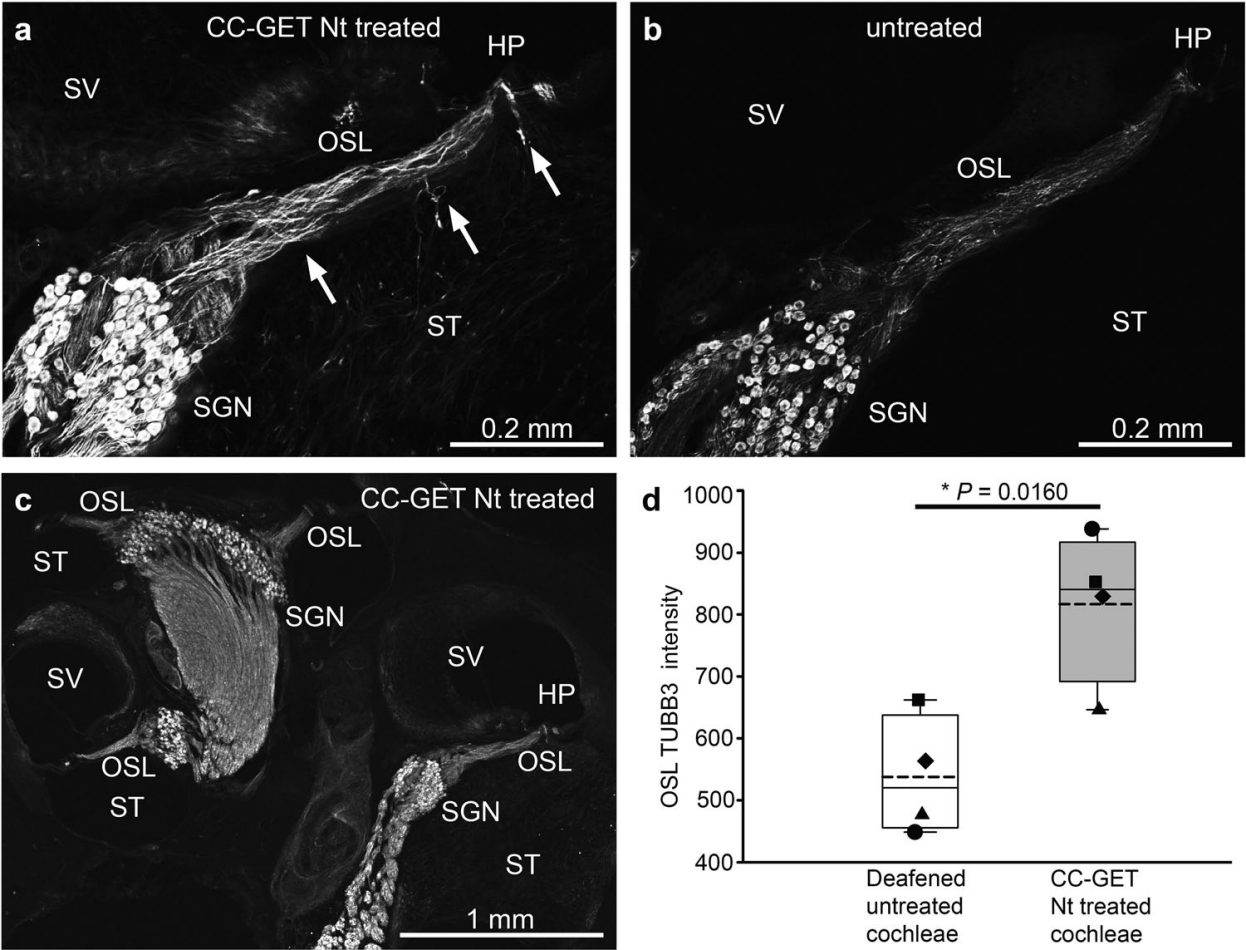
CC‐GET delivery of the clinical pFAR4‐CMVp‐BDNF‐NT3 miniplasmid DNA drives cochlear spiral ganglion neuron (SGN) regeneration in deafened guinea pigs. a) Detail of basal cochlear region showing neurite outgrowth (TUBB3 immunofluorescence) following neurotrophin‐encoding DNA electrotransfer. Arrows indicate ectopic growth of the spiral ganglion neurons (SGN) into scala tympani (ST). b) Untreated opposite cochlea from the same animal showing atrophied SGNs in the same region. c) Low power mid‐modiolar image from (A), showing higher intensity immunofluorescence of the neurites in the osseous spiral lamina (OSL) in the more basal region. d) SGN neurite fluorescence signal (mean pixel intensity) within the distal half of the basal region OSL; 25% and 75% boundaries with 95% limits; individual data from 4 animals at 2 weeks post CC‐GET. Student's paired *t*‐test, one‐tail. HP – habenula perforata, SM – scala media, SV – scala vestibuli.

Confocal immunofluorescence validated the effectiveness of the therapeutic neurotrophin‐encoding miniplasmid in regenerating the peripheral neurites of the spiral ganglion in the deafened guinea pigs, where TUBB3 (β3Tubulin) immunofluorescence intensity in the osseous spiral lamina (OSL) region was compared with batch‐processed untreated opposite cochleae (**Figure** **5**a–c). Dense fascicles of spiral ganglion neurites were evident in the cochleae receiving the neurotrophin gene augmentation, while the untreated cochleae showed a paucity of fibers, consistent with the deafening procedure. The average pixel TUBB3 immunofluorescence intensity in the region of interest (distal 50% of the OSL) of the neurotrophin DNA – treated cochleae was 816.7 ± 61.5, compared to 537.7 ± 48.1 for the untreated control cochleae (*p* = 0.0160, Student's paired *t*‐test, one‐tail) (Figure 5d). In addition, there was a pronounced increase in TUBB3 immunofluorescence in the SGN soma region, reflecting increased translation of the neurofilament protein associated with neurite outgrowth.

### 2.6 Preclinical validation of Long‐Term Stability of the Neurotrophin CC‐GET‐Enhanced Bionic Interface in the Deafened Cat Model

Prior studies in cat and guinea pigs have shown that BDNF‐mediated regeneration of cochlear auditory neurites, albeit with promiscuous outgrowth, can be maintained using chronic stimulation via a CI,^[^ ^25^, ^26^ ^]^ whereas without electrical stimulation, the neurites regress when intracochlear BDNF infusion ceases.^[^ ^27^ ^]^ To evaluate the ability of chronic CI use to promote long‐term stability of the neurotrophin CC‐GET – enhanced bionic interface, a 6‐month longitudinal study of deafened cats was undertaken. The cat cochlea model benefits from the close anatomical semblance with humans, particularly in the basal half of scala tympani.^[^ ^19^ ^]^ This accommodates the transient insertion of zone 1 of the clinical CC‐GET array (electrodes 22–15). Additionally, these animals are able to utilize bilateral CIs for hearing, with associated external speech processors and battery packs accommodated in a jacket, to sustain chronic electrical stimulation.

**Figure 6:**
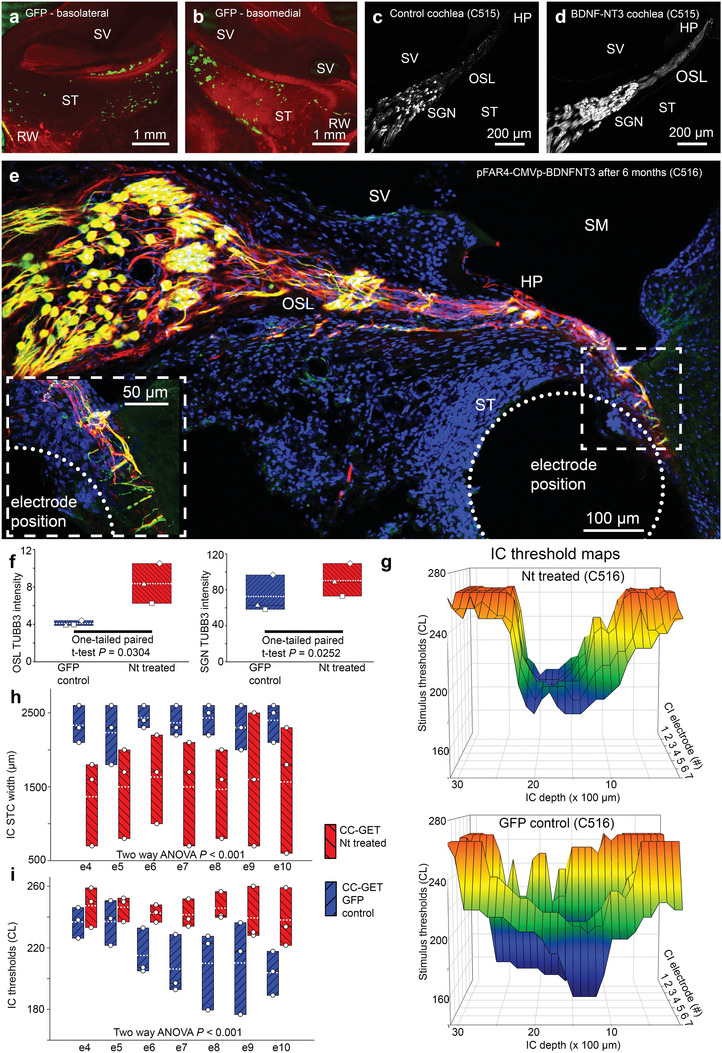
Clinical CC‐GET neurotrophin gene augmentation with chronic cochlear implant (CI) stimulation achieves long‐term rescue of regenerated spiral ganglion neurites and reset of central auditory tuning in the cat inferior colliculus. a,b) Targeting of perilymphatic mesenchymal cells in the basal scala tympani (ST) region confirmed by pFAR4‐CMVp‐eGFP reporter expression; adjacent scala vestibuli (SV) is unlabeled; the GET probe was inserted via the round window (RW) membrane. c,d) Comparison of CC‐GET of control eGFP reporter DNA C), versus pFAR4‐CMVp‐BDNF‐NT3 DNA D) shows long‐term rescue of spiral ganglion neurites (TUBB3 immunolabeling) within the osseous spiral lamina (OSL), supported by sustained (≈6 months) electrical stimulation via the CI. e) Detail of projection of rescued auditory neurites to the region of the CI “closing the bionic interface neural gap” (TUBB3/neurofilament 200 immunolabeling). f) Quantitation of anti‐TUBB3 fluorescence intensity in the OSL region and spiral ganglion neuron somata (SGN) from cryosections (symbols distinguish average data from the three cats). g) Sharper spatial tuning curve (STC) threshold profile recorded through a region of the inferior colliculus driven by the CI in the neurotrophin CC‐GET – treated cochleae (top) compared with input from the control eGFP DNA‐treated cochlea (bottom). h) Spatial tuning curve (STC) widths and i) average stimulus thresholds, show that the cochlear neurotrophin gene augmentation treatment results in an overall reduction in excitability, associated with sharpening of spatial tuning. See also Supporting Information – Methods and Primary Figure Commentary.

Validation of efficient CC‐GET was initially undertaken in one normal‐hearing cat using pFAR4‐CMVp‐eGFP reporter DNA, bilaterally, with fixation of the cochleae after 72 h (**Figure** **6**a,b). These data confirmed that the current pulse profile optimized in the guinea pig study (10 × 100 µs × 50 mA) was effective in cats, and therefore likely to be appropriate for human cochlear gene augmentation treatment. In the pFAR4‐CMVp‐eGFP reporter‐expressing cat cochleae, the GFP‐labeled mesenchymal cells were confined to scala tympani in the basal half of the cochlea, and the highest density of cells was at the basal mid‐turn region, distal from the round window membrane. This demonstrated site‐specific control of the gene electrotransfer matched to the electric field profile established for zone 1 of the clinical CC‐GET device (peak electric field density ≈e19–e18).

Having validated CC‐GET in the cat cochlea using eGFP reporter expression, delivery of pFAR4‐CMVp‐BDNF‐NT3 DNA to one cochlea and pFAR4‐CMVp‐eGFP reporter‐encoding DNA (control) to the opposite cochlea, was undertaken in four cats deafened 1–3 months prior to surgery. Immediately after the electrotransfer current pulse train, the clinical CC‐GET device was withdrawn and replaced with a 14‐electrode CI array controlled by an externalized connector‐coupled Cochlear Nucleus 5 Sound Processor stimulator (Cochlear Ltd.). Thus, the only difference in treatment between the left and right cochleae in each cat was the DNA sequence (randomized and blinded). The DNA electrotransfer utilized a current‐controlled pulsed electric field equivalent to that determined for the preceding guinea pig and cat experiments (Table S2, Supporting Information). Overall, across ten cat cochleae, the average current delivered by 10 × 100 µs pulses for CC‐GET was 34.1 ± 1.6 mA (1 ms combined pulse duration), affording an average charge transfer of 34 µC, via a mean applied voltage of 117.5 ± 2.5 V. On average the clamped resistance equated to 3.55 ± 0.24 kΩ.

The CI arrays were switched‐on 2 weeks postsurgery, and three cats used these devices continuously for hearing for 135–159 days. Functional assessments were performed periodically to measure neural recruitment thresholds from electrically evoked compound action potential (eCAP) recordings (Figure S6, Supporting Information). These data, elicited using monopolar (MP+1) stimulation along the length of the CI array, with recording from adjacent electrodes, showed maintained thresholds of equivalent sensitivity in the auditory nerve across cochleae for both control pFAR4‐CMVp‐eGFP and neurotrophin‐encoding pFAR4‐CMVp‐BNDF‐NT3 DNA CC‐GET. At the 6‐month endpoint, the cats were anesthetized to enable mapping of inferior colliculus neural recruitment via electrical stimulation of the CI arrays for the CC‐GET neurotrophin‐treated cochlea and the CC‐GET‐eGFP‐treated (control) cochlea for each cat. No systemic dispersion of the pFAR4 DNA was detectable by polymerase chain reaction (PCR) analysis in organs, blood, and urine samples (Supporting Information–Methods).

In all three cats, immunofluorescence labeling of fixed, decalcified, and cryosectioned cochleae that had received the CC‐GET neurotrophin gene augmentation treatment 6 months prior, showed the anticipated retention of spiral ganglion neurite regeneration within the osseous spiral lamina projecting in close proximity to the CI array. Consistent with tropic guidance from mesenchymal cell BDNF‐NT3 production, regenerated neurites projected out of the osseous spiral lamina into the proximal aspect of scala tympani, as well as extending past the normal insertion point at the habenula perforata to the lateral spiral ligament, penetrating to the mesenchymal cell lining (Figure 6c–e). The fibrotic infiltration around the CI array evidently acted as a support matrix for the spiral ganglion neurite projections which ended in fine processes within ≈20 µm of the electrodes (Figure 6e inset). This confirmed that the “neural gap” with the CI array could be minimized using the pFAR4‐CMVp‐BDNF‐NT3 neurotrophin CC‐GET technology and that this benefit was maintained with the use of the CI. Tubulin beta 3 class III (TUBB3) immunofluorescence intensity in the peripheral neurites in the osseous spiral lamina was significantly higher in the neurotrophin CC‐GET‐treated cochleae, (neurotrophin‐DNA = 8.35 ± 1.23; eGFP‐DNA = 4.08 ± 0.17; *p* = 0.0304, one‐tailed paired *t*‐test; Figure 5f; Figure S7, Supporting Information). Similarly individual spiral ganglion soma showed significantly increased levels of this microtubule protein in the neurotrophin DNA‐treated cochleae compared with cochleae receiving the pFAR4‐CMVp‐eGFP DNA (mean pixel intensity NT DNA = 90.39 ± 10.54, eGFP DNA = 72.59 ± 12.13; *p* = 0.0252; one‐tailed paired *t*‐test; Figure 5g; Figure S7, Supporting Information). Consistent with neuroprotection associated with neurotrophin treatment,^[^ ^26^ ^]^ the average number of soma in the basal region trended higher in the neurotrophin‐DNA treated cochleae compared with the GFP control cochleae (NT DNA group = 38.33 ± 11.86 neuron soma / SGN ROI; eGFP DNA group 21.25 ± 7.52; *p* = 0.118, one‐tailed paired *t*‐test; *n* = 3 cochleae, each a mean of 4 SGN ROIs/group; Figure S7, Supporting Information).

Imaging failed to detect expression of the eGFP reporter in the control‐side cochlea from each of the cats in the long‐term study, consistent with a fall‐off in transgene expression demonstrated in the guinea pig due to turn‐over of the transfected mesenchymal cells.^[^ ^17^ ^]^ Thus, the retention of regenerated peripheral spiral ganglion neurites arising from a limited period of recombinant BDNF and NT3 expression by the scala tympani mesenchymal cells was most likely achieved through complementary chronic electrical stimulation via the CI. The key differential here is the directed regrowth of the auditory dendrites to the vicinity of the CI electrodes achieved by targeted neurotrophin CC‐GET to the mesenchymal cells proximal to the CI array.

The functional effect of the neural enhancement of the CI interface was validated at the 6‐month endpoint by analysis of spatial tuning curve (STC) widths within the inferior colliculus, recorded under anesthesia using a 32‐channel linear recording array placed in either hemisphere of the IC, and stimulated by both CI arrays using a bipolar stimulation modality, for CI electrodes e4–e10. There was a significant sharpening of the STCs driven by neurotrophin‐treated cochlear input, compared with stimulation via the opposite control cochlear input (Figure 6g–i; Figure S8, Supporting Information). The average STC width with neurotrophin CC‐GET across all 32 IC electrodes driven by 7 CI electrodes was 1519.0 ± 131.6 µm, compared with 2357.1 ± 51.0 µm for the eGFP CC‐GET control cochlear input (*p* < 0.001, two‐way ANOVA) (Figure 6h). This reflects a broad hyperexcitability (lower thresholds) within the IC in response to stimulation of the control cochlear input, whereas the neurotrophin‐treated cochlear drive was more spatially specific. The average threshold across all 32 IC electrodes, driven by the seven control CI electrodes was 217.04 ± 4.66 current level (CL), compared with 243.00 ± 2.43 CL for input from the 7 CI electrodes from the neurotrophin‐treated cochleae (*p *< 0.001, two‐way ANOVA) (Figure 6i). CL is defined by Cochlear Ltd. where, current in µA = 17.5× (100 ^ ^(CL/255)^)

### 2.7 CC‐GET Enables Targeted In Vivo Gene Delivery Within the Brain

The utility of CC‐GET using the bionic array‐based electric field focusing on targeted brain DNA therapeutics was evaluated by in vivo plasmid DNA delivery in guinea pigs. These studies, utilizing nine animals, evaluated expression of a range of reporter constructs, including a tdTomato tagged red‐shifted channel rhodopsin ion channel under the human synapsin (hSYN) promoter (ReaChR; pJL‐hSYNp‐ReaChR‐tdTomato; Figure S1E, Supporting Information), and the cytomegalovirus promoter (CMVp)‐driven GCaMP5g Ca^2+^ reporter (pAG‐CMVp‐GCaMP5g; Figure S1F, Supporting Information). GFP reporter plasmid DNA (pFAR4‐CMVp‐eGFP) was delivered to two sites, the dorsomedial brainstem (nucleus of the tractus solitarius, NTS), and the globus pallidus (GP), deep thalamic region. A second reporter plasmid pMK‐CAGp‐eGFP (Figure S1G, Supporting Information) was delivered to the NTS. The GP brain region is a target for deep brain stimulation bionics to manage debilitating motor symptoms in Parkinson's disease. Gene augmentation to achieve expression of recombinant ion channels at this site is likely to provide therapeutic neuromodulation. Proof of concept for such targeted neuromodulation was established utilizing CC‐GET to achieve light‐pulse activation of GP neurons expressing ReaChR channels, altering Ca^2+^ transients, recorded as changes in GCaMP5g fluorescence in brain slices.

**Figure 7:**
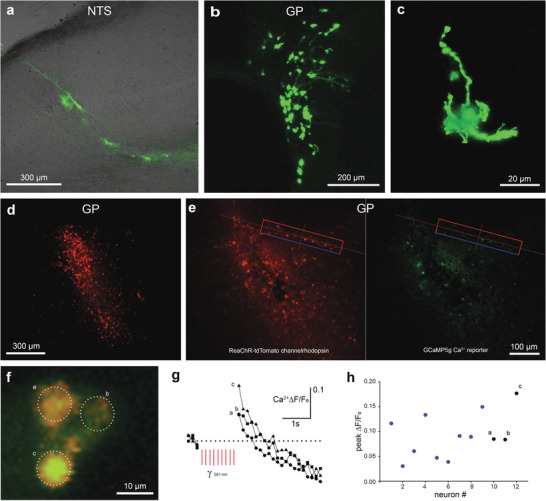
CC‐GET gene delivery to the guinea pig brain achieves targeted optogenetic neuromodulation. A) pFAR4‐CMVp‐eGFP plasmid DNA delivery to the nucleus of the tractus solitarius (NTS) region of the brainstem. B,C) pMK‐CAGp‐eGFP plasmid DNA delivery to the globus pallidus (GP). D,E) Dual delivery of pAG‐CMVp‐GCaMP5g Ca^2+^ reporter + pJL‐hSYNp‐ReaChR‐tdTomato channel rhodopsin plasmid DNAs to GP region (10 × 100 µs × 50 mA pulses). F) Detail of three neurons co‐expressing ReaChR and GCaMP5g. G) Ca^2+^ responses to 561 nm pulsed laser activation of ReaChR channels in the three neurons shown in (F). H) Peak Ca^2+^ responses for 12 neurons. Images from (B) and (C) are from patent ref. [16]. See also Supporting Information–Methods and Primary Figure Commentary.

GFP reporter expression was achieved across both brain region targets (**Figure** **7**a–c). Overall, CC‐GET provided highly reliable gene expression (4/4 animals). The choice of the NTS brainstem region, critical for cardio‐respiratory function, validated the CC‐GET safety profile. CC‐GET was well tolerated with no adverse indicators evident across the 3–7 days until predetermined endpoint. This was supported by the postmortem integrity of brain parenchyma in the regions showing GFP expression in neurons (Figure 7a). These studies demonstrated substantial populations of transfected neurons within both regions (Figure 7a,b). The flexibility of CC‐GET for delivery of cocktails of plasmid DNAs was validated by bilateral targeting of the GP with a combination of pAG‐CMVp‐GCaMP5G DNA and pJL‐hSYNp‐ReaChR‐tdTomato. Both plasmid DNAs achieved neuron‐selective expression, where ReaChr was detected via its fused tdTomato reporter (*n* = 5/5) (Figure 7d–f). ReaChR expression enabled optogenetics‐based neuromodulation of individual GP neurons in brain slices, where GCaMP5g co‐expression reported Ca^2+^ dynamics evoked by 561 nm light‐activation (0.3–1.2 s) (Figure 7g,h). Overall, the peak GCaMP5g ∆F/F_o_ intracellular Ca^2+^ signal after cessation of the red light stimulation was 0.0918 ± 0.0131 above the preceding baseline (3 sample average; *p* = 0.0000223, one sample *t*‐test, *n* = 12 neurons/7 slices).

Table S3 (Supporting Information) provides the current amplitude (range 10–50 mA) and delivered voltages (range 25–90 V) for 10 × 100 µs pulses or 3 × 100 ms pulses for brain CC‐GET, and calculated local resistance determined across the gene delivery array in the presence of plasmid DNA in sucrose carrier. The effectiveness of controlling local brain conductivity was evaluated by comparing resistance measured across the clinical CC‐GET electrode array at baseline (no DNA present), against measurements at the time of GET. Resistance increased by 138% (1.08 ± 0.08 kΩ baseline (*n* = 11) to 2.58 ± 0.29 kΩ (*n* = 8); *p *= 0.0000282; *t*‐test). This reduction in local brain conductivity would significantly increase the focal electric field intensity in the region adjacent to the gene delivery array (as shown in Figure 1), and hence drive efficient electrotransfer of the plasmid DNAs.

### 2.8 Context of CC‐GET Development for Targeted Gene Expression in the Nervous System

The current study advances the translational platform for discrete gene electrotransfer‐based therapeutics within nervous system tissues, demonstrating the utility of electric field focusing achieved via variable configuration of a linear array of electrodes, augmented by suppression of local tissue conductivity (“conductivity‐clamping”). Development of the clinical CC‐GET device gene delivery electrode array enabled precise control of the tissue volume for gene expression of the naked (plasmid) DNA gene payload. The “electro‐lens” effect to expand, or contract, the field of transfected cells was achieved by changing the separation of the linear cathode and anode electrode elements. Local control of tissue conductivity by displacement of the physiological ionic milieu with nonionic sucrose carrier biased the local current flow between the CC‐GET probe electrodes to the polyanionic DNA species, amplifying local electric field strength for a given charge delivery.

The local extracellular (field) voltage provides the driving force for DNA transfer, but central to this is the change in voltage with distance (electric field strength) at the cellular level. In our in vitro HEK293 cell modeling, the threshold for plasmid DNA CC‐GET was ≈120 V cm^−1^ (Figure 2). The linear electrode array used for close‐field CC‐GET maximizes this local field potential gradient, evident from the electric field maps, where field strength can be maximum close to the null point (zero voltage) zone between the anodes and cathodes (Figure 2 and ref. [12]). With conventional open field GET, because of the relatively high extracellular tissue conductivity, a large amount of current is needed to achieve suprathreshold field strengths. Examples include applications across a range of clinical electroporation devices driving multi‐electrode configurations for intradermal delivery of plasmid DNA vaccines subcutaneously, requiring 400–500 mA with pulse widths of 52–60 ms (reviewed by Broderick and Hueau^[^ ^28^ ^]^); equivalent to ≈25 mC of charge per pulse (2–3 pulses). Such high current fluxes lead to tissue damage associated with local ohmic heating and irreversible electroporation.^[^ ^18^ ^]^ This was illustrated in the present study with delayed application of propidium iodide following GET with saline carrier at 3 × 100 ms × 35 mA pulses (10.5 mC), where the HEK293 cells exhibited fluorescence in a margin adjacent to the gene delivery array placement.

Across studies involving electroporation‐mediated DNA vaccine delivery to hundreds of human subjects, pain is a notable side‐effect, taking several minutes to subside.^[^ ^28^ ^]^ In comparison, the threshold charge delivery per pulse for CC‐GET incorporating the sucrose carrier (3 × 5 mA × 100 µs pulses in HEK293 cell monolayers, as shown in Figure S3, Supporting Information; US patent ref. [16]), was 0.5 µC per pulse (50 000 times less charge). For perspective, this is only marginally greater than the charge delivered per pulse in CI devices at continuous neural stimulation at rates in the kHz range for routine hearing prosthesis function.^[^ ^29^ ^]^ The CC‐GET refinements applied in vivo achieved functionally comparable gene expression in the cochlea at ≈1000‐fold less charge compared with the original bionic array‐based close‐field GET study by Pinyon et al.^[^ ^11^ ^]^ This speaks to the efficiency and safety of the CC‐GET technology. Further, the GET of plasmid DNA is highly permissive with regard to packaging size, with the present study demonstrating high expression efficacy across seven plasmids ranging in size from 2.83 to 7.68 kb, in the MDa range.

### 2.9 Translational Pathway for Clinical CC‐GET‐Mediated Neurotrophin Gene Augmentation to Enhance Bionic Hearing

The CC‐GET probe included micro‐fluidics for delivery of the humanized bicistronic expression cassette encoding the BDNF & NT3 genes within a pFAR4 miniplasmid, eliminating the potential for dissemination of antibiotic resistance genes associated with standard plasmid vectors, and satisfying the European Medicines Agency requirements for GET.^[^ ^21^ ^]^ The delivery of this therapeutic DNA and sucrose formulation via insertion of the CC‐GET device probe through the cochlear round window displaced the perilymph, reducing local conductivity in the tissue around four‐fold across guinea pig and cat experiments, with commensurate reduction in the electric current levels needed to achieve a therapeutic level of gene expression. As such, the capability of CC‐GET for gene augmentation applications was advanced to a clinic‐ready technology aiming to improve hearing outcomes in CI recipients by using precise delivery of neurotrophins directing auditory nerve regeneration toward the CI array, closing the neural gap and thereby improving the bionic interface.

The safety and efficacy of the CC‐GET neurotrophin treatment in the cochlea was validated in vivo in the deafened guinea‐pig model, demonstrating rapid regeneration of the auditory nerve, while the chronic studies in the neonatally‐deafened cat model validated the long‐term safety and viability of the treatment. This outcome highlights the broad potential for neurotrophin‐based CC‐GET to regenerate peripheral nerves. These studies, along with subsequent good manufacturing practice (GMP) production of the clinic‐ready CC‐GET device, and the humanized neurotrophin‐encoding DNA therapeutic molecule, supported the registration of a first‐in‐human phase I/IIa clinical trial of neurotrophin CC‐GET gene augmentation treatment during CI surgery through the Australian Therapeutic Goods Administration^[^ ^30^ ^]^ (currently underway). It is notable that gene augmentation treatments using (biologically inert) naked DNA are not subject to gene technology regulatory oversight in Australia, but rather lie within the conventional medical device‐small molecule therapeutics clinical trial framework.

The use of this CC‐GET device to deliver the pFAR4‐BDNF‐NT3 miniplasmid resulted in highly reproducible directed regeneration of the cochlear auditory nerve fibers in both animal models. Thus, the key benefit here of the CC‐GET targeted delivery to the cochlear mesenchymal cells adjacent to the subsequently implanted CI array is the establishment of a local tropic signaling gradient where recombinant BDNF is able to establish guidance via the activation of its cognate neurotrophic tyrosine kinase receptor type 2/TRKB receptor on the spiral ganglion neurons, with likely downstream signaling via G_αq_‐ phospholipase Cγ (PLCγ)‐ phosphotidylinositol 4,5‐bisphosphate (PIP_2_)‐ diacylglycerol (DAG) second messenger signaling of canonical transient receptor potenital 3 (TRPC3) channels,^[^ ^31^ ^]^ which are known to be expressed by the SGNs.^[^ ^32^ ^]^ The CC‐GET‐mediated recombinant BDNF production is likely to have dominated the spiral ganglion neurite regeneration, as this was most effectively secreted in the in vitro HEK293 cell model, and BDNF levels in the bulk guinea pig perilymph samples of the neurotrophin‐CC‐GET study were four times higher than the background, while the perilymph NT‐3 levels in vivo were not significantly greater than the untreated cochlea reference level. In the cochlea, the sensory hair cells are the principal source of BDNF.^[^ ^33^ ^]^ NT3 is associated with the supporting/glia‐like cells in the cochlea, alongside inner hair cells, and facilitates auditory synapse recovery after noise trauma in the basal (high‐frequency) encoding region.^[^ ^34^, ^35^ ^]^ The deafening procedure leads to the loss of these organ of Corti sensory hair cells and supporting cells, disrupting BDNF/NT3 production, leading to retraction of the SGN neurites and atrophy of the somata, while the centrally connected auditory axons remain viable, albeit with some SGN loss.

The SGN nerve regeneration was transient in the deafened guinea pigs. This most likely arose from fall‐off in the recombinant neurotrophin secretion as the targeted mesenchymal cells lining the scala media compartment turned‐over.^[^ ^17^ ^]^ In previous cat and guinea‐pig studies, chronic electrical stimulation via the CI devices achieved maintenance of BDNF‐infusion‐retrigerated cochlear spiral ganglion neurites.^[^ ^25^, ^36^ ^]^ A key distinguishing feature of preceding deafened cochlear preclinical studies using direct infusion, or viral‐vector‐mediated delivery of BDNF or NT3, was the unintended ectopic over‐extension of the neurites that occurred.^[^ ^25^, ^37^, ^38^ ^]^ This can thwart the utility of CIs, where local stimulation of sub‐populations of nerve fibers is required to match the tonotopic mapping of the cochlea that underlies pitch perception. In the present study, the spiral ganglion neurite outgrowth was discrete, and with chronic stimulation via the CI in the cat model, the fibers remained in close proximity (<20 µm; Figure 5e) to the electrode array for the ≈6‐month duration of the study. This enhanced neural interface significantly reset spatial tuning within the inferior colliculus.

The plasticity of the auditory brainstem pathways underpins the restoration of hearing with the “bionic ear.”^[^ ^39^ ^]^ In the deafened cat model, it has been reported that with deafening, with or without chronic electrical stimulation via a CI, intrinsic plasticity in the primary auditory cortex lends broad zones of activation, associated with local circuit disinhibition.^[^ ^40^ ^]^ In the inferior colliculus of adult cats, the STC widths broaden with deafening, and to an even greater extent in neonatally deafened cats,^[^ ^41^ ^]^ the model used in the present study. Nonspecific chronic electrical stimulation via the CI can cause further de‐tuning of the cochleostomy at this level.^[^ ^41^ ^]^ In the present study, the cats experienced sustained use of their bilateral CIs (with and without the neurotrophin gene augmentation), with clinical speech processors enabling environmentally relevant sound encoding and communication for many months. This natural dynamic auditory stimulation is evidently matched to the restoration of central auditory circuit functionality, where with the improvement of the neural interface as a result of neurotrophin gene augmented CI treatment (enhanced auditory drive), there was a significant rescue of spatial mapping of hearing coding, which would support upstream improvement in thalamic and cortical auditory circuit performance. This may be at the local synaptic level within the inferior colliculus, where the contralateral drive to the same neural field via the cochlea receiving only the eGFP control DNA exhibited broad STC widths. This contributes insight into the plasticity of central auditory processing that likely underpins the progressive improvement in hearing performance that occurs in the months following CI surgery.^[^ ^42^ ^]^

### 2.10 Extension of CC‐GET to Precision Gene Transfer Targeting Within the Brain

Gene therapy in the broadest sense of DNA/RNA‐based therapeutics is increasingly being pursued to address CNS neurological disorders.^[^ ^43^, ^44^ ^]^ The extension of the utility of CC‐GET‐based focal gene augmentation to brain therapeutics was demonstrated by targeted gene expression across a range of guinea pig brain regions and validation of targeted neuromodulation via optogenetics. Currently, there are more than 179 active studies in clinical trials for CNS disorders, including a lentiviral vector‐based study for drug‐resistant intractable epilepsy and more than ten lentiviral and AAV‐2‐based gene therapy clinical trials directed to Parkinson's Disease (PD).^[^ ^45^, ^46^ ^]^ Contrasting with the limited spatial targeting of viral vector‐based gene delivery due to sustained transduction activity as viral particles disperse, pulsed electric field‐based “naked” DNA electrotransfer using conductivity‐clamping has the evident potential, clearly validated here, to achieve superior spatial control of gene expression and resolve these other challenges, as well as being cost‐effective and faster to implement. However, no CNS gene therapy clinical trials have adopted clinical electroporator‐based gene transfer; reflecting the incompatibility of electrode invasiveness in clinical GET devices, and the tissue‐damaging current pulses and associated charge transfer needed for DNA electrotransfer with those devices. Across brain regions, in cases where plasmid DNA‐based gene expression has been achieved in vivo in the adult mammalian brain via GET, “micro‐electroporation” has achieved limited neuronal expression across a range of brain regions in preclinical models, utilizing single or paired needle electrodes. This includes lateral geniculate nucleus targeting in mice and cats,^[^ ^47^ ^]^ suprachiasmatic region in hamsters,^[^ ^48^ ^]^ dual small interfering RNA (siRNA) and plasmid reporter DNA delivery into rat anterior cingulate cortex.^[^ ^49^ ^]^ It is notable that brain electrolytic lesions are reported in the region of the GET, particularly close to the electrodes (highest field strength zone) at high voltage–current levels.^[^ ^47^, ^50^ ^]^ Use of closely apposed electrophysiology recording electrodes, as GET electrodes, stereotaxically positioned within the mouse hippocampus enabled delivery of a β‐galactosidase reporter plasmid (saline carrier with low current pulses; 125 µA x 50 ms; 6 µC charge), consistently achieving small fields of transformed neurons.^[^ ^51^ ^]^ A similar approach using a single electrode with a needle as the anode return as well as providing local delivery of a range of plasmid DNAs, achieved consistent focal expression within neurons, or astrocytes (DsRed reporter driven by a glutamate‐aspartate transporter (GLAST) promoter) within the striatum in adult mice, with low voltage pulses (25–35 V × 50 ms). Minimum tissue injury was observed outside of the immediate region of the electrode and delivery needle. These preceding studies highlight the requirements for low current amplitudes and local delivery of the DNA for focal GET in the brain.

In the present study, the combination of electrode array‐based electric field “lens” focusing and conductivity – clamping enabled the clinical CC‐GET device to achieve highly reproducible targeted delivery of plasmid DNAs to transduce substantial fields of neurons within the adult guinea pig brain. The inclusion of microfluidics to deliver the sucrose‐based non‐ionic carrier and plasmid DNA within the suprathreshold electric field to locally reduce conductivity and thereby amplify the electric field was effective, as the resistance in the close‐field was increased by 138%.

The dual neuronal expression of plasmid DNAs encoding red‐shifted channel rhodopsin (ReaChR) and the GCaMP5g Ca^2+^ reporter in the globus pallidus brain region enabled “proof of concept” for optogenetics‐based neuromodulation. In PD, this is the site of hyperactivated premotor neurons, due to disinhibition through loss of dopaminergic substantia nigra input.^[^ ^46^ ^]^ Current management of the PD‐associated movement dissociation (dyskinesia) includes the use of bionic array‐based DBS, as the standard of care for patients associated with loss of effectiveness of L‐DOPA treatment.^[^ ^52^ ^]^ DBS uses cochlear implant‐like electrode arrays to provide continuous stimulation to suppress neuronal activity within the globus pallidus internus and subthalamic nuclei (where for example, the device may operate at up to 250 Hz with 25 mA × 450 µs pulses (≈11 µC of charge per pulse),^[^ ^20^, ^53^ ^]^ which for perspective, is >20× the CC‐GET threshold). Based on the findings from the present study, spatially‐controlled CC‐GET could be used to safely target this brain region for neuromodulation via recombinant ion channel expression, to complement or replace DBS, including the use of optogenetics strategies in combination with bionic arrays (a feature emerging in the CI field).^[^ ^54^, ^55^ ^]^ Alternatively, emerging technologies for intrinsic regulation of gene dose of recombinant K^+^ channels, such as demonstrated preclinically in a DNA therapeutics model for epilepsy utilizing Kv1.1 channels,^[^ ^56^, ^57^ ^]^ could broadly enable neuromodulation via CC‐GET to treat focal neurological disorders and brain pathophysiology. CC‐GET could also enable local expression of neurotrophins, particularly BDNF, which provide neuroprotection from the subacute phase of secondary brain injury expansion following stroke.^[^ ^58^ ^]^ Such approaches may be extended to RNA‐based therapeutics, which would address the necessity to manage the duration of treatment in neurons, which being terminally differentiated, could potentially express therapeutic plasmid DNAs indefinitely. DNA therapeutics engaging immunological mechanisms are also emerging for brain disorders. Preclinical in vivo models using AAV delivery of interleukin 2 encoding DNA in the brain stimulate the expansion of resident regulatory T cells that suppress neuroinflammatory responses associated with stroke, traumatic brain injury, multiple sclerosis, and cognitive decline.^[^ ^59^, ^60^ ^]^ There is also clear potential to develop CC‐GET to achieve recombinant cytokine expression targeting intratumoral therapeutics for brain tumors such as glioblastoma multiforme (GBM). Such DNA/RNA therapeutics are relevant to the broad range of GBM treatment options currently in preclinical evaluation, or clinical trial, including viral vector‐delivered suicide genes such as thymidine kinase, and the use of vascular endothelial growth factor receptor proteins to inhibit tumor angiogenesis (reviewed by refs. [61, 62]).

## 3 Conclusion

The technical development and translational pathway for the clinic‐ready CC‐GET medical device is outlined here. The precision delivery of a human‐gene therapy optimized plasmid DNA encoding the BDNF and NT‐3 neurotrophins to the cochlea in deafened animal models drove auditory nerve regeneration that achieved sustained improvement in hearing performance with cochlear implants. This established the case for clinical trial registration for a first‐in‐human phase I/IIa study. The biophysical properties of the conductivity‐clamping enhanced focused electric field GET were found to match the needs for focal brain therapeutics. The CC‐GET technology provided control of DNA delivery titer, matched to targeted spatial expression mapping, brain cell‐type selectivity, safety, and durability. Proof of concept was achieved for photonics‐based neuromodulation via channelrhodopsin expression in a brain region relevant to the treatment of Parkinson's disease. This capability aligns with the unmet need for treatment of a range of focal neurological disorders and brain oncology applications. Overall, there are evident advantages to CC‐GET‐based gene delivery, these include a lack of size constraints on gene construct packaging, and a lack of immunogenicity of naked DNA, which is a major drawback of viral vector‐based delivery. This provides an improved safety profile and lowered regulatory barriers, with faster and lower‐cost development. The key feature of instantaneous “fixing” of the field of gene expression to the brief period of delivery of the pulsed electric field overcomes the challenge inherent to other gene delivery systems. These properties are ideal for targeted gene delivery across the central and peripheral nervous systems, and muscle, and a breadth of emerging applications for nucleic acid‐based gene therapeutics. While the current clinical CC‐GET device design is adapted to provide flexible conformation to soft tissue targets, the technology lends itself to the development of complementary minimally invasive electro‐lens probes for directed deep tissue targeting of DNA/RNA delivery.

## 4 Experimental Section

### Guinea Pig Cochlear CC‐GET

The guinea‐pig model was used to establish in vivo validation of gene expression using the antibiotic‐free pFAR4 gene vector, including establishing duration of expression in the targeted cochlear scala tympani mesenchymal cells and levels of recombinant neurotrophins (BDNF and NT3). All guinea pig experiments were conducted following protocols approved by the UNSW Sydney Animal Care and Ethics Committee (ACEC #: 13/79B; 15/09B; 16/172A), under the Australian National Health and Medical Research Council (NHMRC) guidelines for the care and use of animals for scientific purposes.^[^ ^63^ ^]^ Guinea pig cochlea CC‐GET was undertaken as previously described.^[^ ^11^, ^15^ ^]^ Young adult guinea pigs of both sexes were anesthetized using isoflurane, and analgesia was provided (buprenorphine (15 µg kg^−1^ i.m., Temgesic, Reckitt Benckiser) and medetomidine hydrochloride (175 µg kg^−1^ i.m., Domitor, Pfizer Animal Health)) in addition to lignocaine HCl (1% solution; Troy Laboratories), for local pain relief. An incision was made above the mastoid process and a hole was drilled to expose the round window of the cochlea. The CC‐GET device developed for clinical application (manufactured by Cochlear Ltd.) was preloaded with pFAR4 DNA resuspended in 10% sucrose, 0.5 mm NaOH at 2 ug µL^−1^. This bionic array‐derived device was modified from a CI622 type half‐banded 22‐electrode array configured for dual eight‐electrode electric field zones (e22–e15; e14 −7 in “tandem ganged configuration”; after^[^ ^11^ ^]^) and incorporated a microfluidics channel for delivery of the plasmid DNA via the tip of the array. The most distal eight electrodes were inserted into the basal turn of scala tympani through a perforation made in the round window membrane and 50 µL was infused over 5 min into the left cochleae only, delivered via a syringe pump. Immediately after DNA infusion the electrical pulse train (10 × 100 µs × 50 mA square wave pulses; 400 µs interpulse interval) was delivered to the clinical CC‐GET array via an isolated stimulator (Digitimer DS5) using a custom‐built waveform controller and monitoring software which captured the voltage required to deliver each current pulse (enabling measurement of the close‐field resistance/conductivity‐clamping in the tissue). Experiments that utilized the pFAR4‐eGFP plasmid were conducted in normal‐hearing animals. Experiments that used the pFAR4‐CMVp‐BDNF‐NT3 plasmid were conducted in guinea pigs that had been deafened using a combination of intravenous furosemide and subcutaneous kanamycin as previously described.^[^ ^11^, ^15^ ^]^ Details of guinea pig tissue collection and analysis can be found in the Supporting Information.

### Auditory Nerve Regeneration in the Cat

The neonatal deafened cat model was used for long‐term studies evaluating the utility of CC‐GET electrotransfer of the clinical neurotrophin encoding DNA for cochlear auditory nerve regeneration and retention with chronic electrical stimulation via CIs. All cat experiments were conducted following protocols approved by the Bionics Institute Animal Research and Ethics Committee and care and practice followed the NHMRC guidelines.^[^ ^63^ ^]^ Neonatal cats were deafened via daily subcutaneous injections of neomycin trisulphate (60 mg kg^−1^) over 3 weeks from birth, which was confirmed by auditory brainstem response recordings as previously described.^[^ ^40^ ^]^ Details of the cat CI surgery and pFAR4‐CMVp‐BDNF‐NT3 CC‐GET procedure and recording of inferior colliculus multi‐channel field recordings mapping cochleotopy, driven by either the neurotrophin gene therapy CI array or the contralateral control array, are described in the Supporting Information.

### CC‐GET Targeted DNA Delivery Within the Guinea Pig Brain

The utility of CC‐GET for targeted gene expression in the brain, including proof of concept for neuromodulation, was evaluated in vivo in the guinea pig. All guinea pig experiments were conducted following protocols approved by the UNSW Sydney Animal Care and Ethics Committee (ACEC #: 16/172A) and NHMRC guidelines as above. Animals were anesthetized as described for the guinea pig cochlea CC‐GET experiments. Guinea pigs were placed on a heated mat in a small animal stereotaxic frame (model 940, Kopf Instruments), and the scalp was shaved and cleaned with iodine and ethanol wipes. An incision was made through the skin down the midline of the head on top of the skull and retractors were used to keep the incision open. Burr holes were drilled through the skull above the target region. The dura was punctured with a 29 G needle and the CC‐GET array was inserted to a depth of 5 mm. The DNA solution was delivered through the array via a syringe microperfusion pump (IM‐1, Narashige). Plasmid DNAs: pFAR4‐CMVp‐eGFP, pJL‐hSYNp‐ReaChR‐tdTomato, pAG‐CMVp‐GCaMP5g, or pMK‐CAGp‐eGFP were delivered in 10% sucrose containing 0.5 mm NaOH at a total concentration of 2 µg µL, 1 µg µL each plasmid where cocktails of two plasmids were used. Following the infusion of DNA, pulse trains were delivered to the CC‐GET array as described for the guinea pig and cat cochlea experiments. Pulse amplitude was set to either 10, 25, or 50 mA, using either 3 × 100 ms or 10 × 100 µs pulses (see Table S3, Supporting Information). For optogenetic neuromodulation‐based Ca^2+^ imaging, three days post CC‐GET the brain was extracted immediately following euthanasia, placed into ice‐cold sucrose‐modified artificial cerebral spinal fluid and cut into 300 µm sections using a vibratome (VT1200S, Leica). Brain slices were imaged on a confocal microscope (LSM710, Zeiss) with ReaChR channelrhodopsin activation evoked using a 561 nm DPSS laser and GCaMP5g Ca^2+^ dynamics detected using 488 nm argon ion laser excitation. For GFP and tdTomato reporter readouts, animals were systemically perfused with phosphate‐buffered 4% paraformaldehyde prior to tissue collection, and slices were imaged using the same Zeiss microscope with 488 nm excitation for GFP and 561 nm excitation of tdTomato.

### Statistical Analysis

Statistical significance was determined using alpha value = 0.05; no data were excluded as outliers (evaluated using the Grubb's test (ESD, extreme studentized deviate; PRISM)). Average data values are reported as mean ± standard error of the mean (s.e.m.), with experimental numbers reported. Statistical analysis was conducted, and graphs were generated using SigmaPlot statistical analysis software (Systat Software Inc. V.14). Parametric statistical assessments were applied following confirmation of normal distribution and equal variance (*t*‐tests (single sample, paired and unpaired; one and two‐tailed) and one way and two‐way ANOVA, including repeated measures where relevant). Data that failed the normal distribution and equal variance tests were transformed and analyzed using nonparametric ranked comparisons (one‐way ANOVA on ranks). Post hoc comparisons on ANOVA used the Holm–Sidak method.

## Conflict of Interest

The authors declare no conflict of interest.

## Author Contributions

G.H., J.L.P., N.L., M.K., R.S., C.S.B., C.Mc., A.W., J.F., and J.F.P. performed conceptualization. G.H., J.L.P., N.L., E.C., J.F., A.W., R.S., Y.L.E., P.C., A.A., M.K., C.J.B., J.M.P., J.L., R.G., D.S., and C.M. performed methodology. J.L.P., G.H., E.C., C.J.B., D.H., G.J., A.A., J.M.P., A.W., J.F., Y.L.E., P.C., D.S., and C.M. performed investigation. J.L.P., G.H., A.A., C.J.B., G.J., and E.C. performed visualization. G.H., N.L., M.K., J.L.P., G.J., R.S., C.Mc., C.S.B., A.W., J.F., Y.L.E., P.C., J.F.P., A.A., W.L., D.Mc., and D.S. performed funding acquisition. G.H. and J.L.P. performed project administration. G.H. performed supervision. J.L.P., G.H., and G.J. wrote original draft. J.L.P., G.H., G.J., C.J.B., N.L., A.A., M.K., E.C., D.H., A.W., J.F., R.S., C.S.B., D.Mc., C.Mc., J.M.P., W.L., J.L., R.G., Y.L.E., P.C., J.F.P., D.S., and C.M. wrote, reviewed, and edited.

## Supporting information

Supporting Information

## Data Availability

The data that support the findings of this study are available in the supplementary material of this article.
