# Supplementary material for: Gene Electrotransfer via Conductivity‐Clamped Electric Field Focusing Pivots Sensori‐Motor DNA Therapeutics: “A Spoonful of Sugar Helps the Medicine Go Down”
Source: Adv Sci (Weinh). 2024 Jun 14;11(30):2401392. doi: 10.1002/advs.202401392 (PMC11321635; doi:10.1002/advs.202401392)
Supplement: Supplementary file 1 — Supporting Information [file ADVS-11-2401392-s001.pdf]

ADVANCED SCIENCE
Open Access

Supporting Information

for *Adv. Sci.*, DOI 10.1002/advs.202401392

Gene Electrotransfer via Conductivity-Clamped Electric Field Focusing Pivots Sensori-Motor DNA Therapeutics: "A Spoonful of Sugar Helps the Medicine Go Down"

*Jeremy L. Pinyon, Georg von Jonquieres, Edward N. Crawford, Amr Al Abed, John M. Power, Matthias Klugmann, Cherylea J. Browne, David M. Housley, Andrew K. Wise, James B. Fallon, Robert K. Shepherd, John Y. Lin, Catherine McMahon, David McAlpine, Catherine S. Birman, Waikong Lai, Ya Lang Enke, Paul M. Carter, James F. Patrick, Robert D. Gay, Corinne Marie, Daniel Scherman, Nigel H. Lovell and Gary D. Housley**

Supporting Information

GENE ELECTROTRANSFER VIA CONDUCTIVITY-CLAMPED ELECTRIC FIELD FOCUSING PIVOTS SENSORI-MOTOR DNA THERAPEUTICS; '*A SPOONFUL OF SUGAR HELPS THE MEDICINE GO DOWN*'

Jeremy L. Pinyon[1,2], Georg von Jonquieres[1], Edward N. Crawford[1], Amr Al Abed[1], John M. Power[1], Matthias Klugmann[1], Cherylea J. Browne[1,3], David M. Housley[1], Andrew K. Wise[4], James B. Fallon[4], Robert K. Shepherd[4], John Y. Lin[5], Catherine McMahon[6], David McAlpine[6], Catherine S. Birman[6,7,8,9], Waikong Lai[9], Ya Lang Enke[10], Paul M. Carter[10], James F. Patrick[10], Robert D. Gay[10], Corinne Marie[11,12], Daniel Scherman[11,13], Nigel H. Lovell[1] and Gary D. Housley[1*]

Affiliations:
[1]Translational Neuroscience Facility, Department of Physiology, School of Biomedical Sciences, Graduate School of Biomedical Engineering, Tyree Institute for Health Engineering (IHealthE), UNSW Sydney, NSW 2052, Australia
[2]Charles Perkins Centre, School of Medical Sciences, Faculty of Medicine and Health, University of Sydney, NSW 2006, Australia
[3]Medical Sciences, School of Science, Western Sydney University, NSW 2560, Australia
[4]Bionics Institute, St Vincent's Hospital, Melbourne, Australia; Department of Medical Bionics; Department of Otolaryngology University of Melbourne, Melbourne 3002, Australia
[5]Tasmanian School of Medicine, University of Tasmania, Hobart, TAS 7001, Australia
[6]Faculty of Medicine and Health Sciences, The Hearing Hub, Macquarie University, Sydney 2109, Australia
[7]Faculty of Medicine and Health, University of Sydney, Sydney, NSW 2006, Australia
[8]Department of Otolaryngology, Royal Prince Alfred Hospital, Camperdown, NSW 2050, Australia
[9]NextSense, Royal Institute of Deaf and Blind Children, Gladesville, NSW 2111, Australia
[10]Cochlear Limited, 1 University Avenue, Macquarie University, NSW 2109, Australia
[11]Université Paris Cité, CNRS, Inserm, UTCBS, F-75006 Paris, France
[12]Chimie ParisTech, Université PSL, 75005 Paris, France
[13]Fondation Maladies Rares, 96 rue Didot, 75014 Paris, France

Corresponding Author*
Gary D. Housley
School of Biomedical Sciences
Faculty of Medicine & Health
UNSW Sydney
NSW 2052
Australia
E: g.housley@unsw.edu.au
T: +61404235180

Advanced Science Pinyon *et al.*, Supporting Information (doi 10.1002/advs.202401392)

Supporting Information Content Summary

Materials and Methods

Electric Field Recordings

HEK293 cell monolayer modelling

Cochlear implant surgery and pFAR4-BDNF-NT3 CC-GET

Cat inferior colliculus recordings

Plasmid DNA

Guinea pig tissue collection and analysis

Cat tissue collection and analysis

Montecarlo analysis of CC-GET electric field thresholds

Measurement of recombinant BDNF and NT3 by ELISA

PCR probe for tissue dispersion of neurotrophin miniplasmid

Figures

Fig. S1: Plasmid DNA maps

Fig. S2: Increase in area of transfected HEK293 cells expressing GFP following CC-GET at equivalent applied voltage using sucrose DNA carrier solution (conductivity-clamping) compared with saline carrier; efficiency and cell injury prevention

Fig. S3: Determination for threshold for reporter plasmid expression with clinical CC-GET DNA electrotransfer using low conductivity-clamping via application of 10% sucrose DNA carrier solution

Fig. S4: Longitudinal study of CC-GET - directed expression of the pFAR4 CMVp-eGFP reporter plasmid to the cells lining the guinea pig cochlea basal region perilymphatic scalae

Fig. S5: Cochlear perilymph recombinant neurotrophin levels in deafened guinea pigs two weeks after CC-GET delivery of clinical pFAR4-CMVp-BDNF-NT3 DNA

Fig S6: Electrically evoked compound action potential (ECAP) thresholds after CC-GET delivery of neurotrophin or control (eGFP) DNA to the cochleae of deafened cats, supporting long-term hearing using bilateral cochlear implants

Fig. S7: Sustained regeneration of the peripheral spiral ganglion neurites in the cochleae of deafened cats supported by chronic cochlear implant stimulation following CC-GET delivery of neurotrophin-encoding DNA

Fig. S8: Sharpening of inferior colliculus spatial tuning curves with cochlear CC-GET delivery of BDNF – NT3 neurotrophin DNA followed by chronic cochlear implant stimulation in the deafened cat

Tables

Table S1: Pulse parameters and resistivity for *in vivo* guinea pig cochlea DNA electrotransfer using the clinical CC-GET device
Table S2: Pulse parameters and resistivity for *in vivo* cat cochlea DNA electrotransfer using the clinical CC-GET device
Table S3: Pulse parameters and resistivity for *in vivo* DNA electrotransfer in guinea pig brain using the clinical CC-GET device

Primary figure commentary (Figs. 1, 2, 4, 6 and 7)

Supporting Information - Materials and Methods

Electric Field Recordings

To establish the effect of conductivity - clamping on the GET electric field, voltage potentials were measured adjacent to the GET array (modified from an 8 electrode research cochlear implant (CI) array, Cochlear Limited, Australia, part number Z60274) (after [1]). This was done in either non-conductive (10% sucrose), or conductive (0.9% sodium chloride) solutions, with or without 2 mg/ml salmon sperm DNA sheared to an average length of 2.0 kb (Merk). The GET array electrodes were driven in the 'tandem' configuration, with four ganged anodes and four ganged cathodes, as shown in Figure 1A, using either a DS5 isolated stimulator (Digitimer) for fixed recording electrode position experiments (Figure 1A, B), or an A-M Systems 2200 stimulus isolator for field mapping (Figure 1C, D) to drive 100 ms square wave pulses. Pulses were controlled and voltage and current outputs monitored (five channels at 100 kHz sampling rate) using a Digidata 1440 interface (Axon Instruments) with Clampex software (version 10.2.0.12; Axon Instruments). A custom-built electrode monitoring system was used to provide independent measurements of voltage in the field, and both voltage and current applied at the electrodes. The recording electrode was a custom platinum fine tip electrode, referenced to a platinum ground electrode positioned distal to the GET array and the generated electric field. Measurements were taken at ~ 95 ms during the 100 ms pulses. Electrodes were positioned using a translational stage (SliceScope Pro 6000; Scientifica, East Sussex, UK) and micromanipulator (ROE-200; Sutter Instrument Company, Novato, CA, USA) with 0.5 mm resolution steps.

HEK293 cell monolayer modelling

The HEK293 cell monolayer was used as a high-throughput *in vitro* model to establish a reporter-based biomarker of the suprathreshold electric field for GET. Adherent HEK293 cells were maintained and prepared for transfection experiments by seeding onto 18 mm round glass coverslips ~ 24 hr prior to transfection, which was undertaken at ~50% cell confluence, following protocols previously published ([1-3]). Plasmid DNA resuspended at 2 mg/ml was delivered to the HEK293-coated coverslips in 20-30 µl of 10% sucrose, with or without 0.5 mM NaOH, or saline, with or without 50 mM Tris buffer, or 9% sucrose + 0.09% NaCl + 0.5 mM NaOH. Electric pulse trains were delivered via a DS5 (Digitimer) isolated stimulator using a custom-built waveform control and monitoring software. Transfected coverslips were imaged

2-4 days after GET on a LSM710 confocal microscope (Zeiss) using a 488 nm argon ion laser for GFP and a 561 nm DPSS laser for mCherry. For larger fields of transfection, multiple overlapping tiled images were captured and assembled prior to analysis. Boundaries around the region of transfected cells were delineated and the area of transfection or total fluorescence (sum pixel intensity) calculated using ImageJ (US NIH).

Cochlear implant surgery and pFAR4-BDNF-NT3 CC-GET in cats

At ~ 3 months of age, cats underwent CC-GET prior to bilateral implantation of a HL14 CI electrode array (Cochlear Ltd.). The surgery was performed as previously described [4] with the exception that prior to implantation of each cochlea CC-GET was undertaken using a double-insertion technique where the clinical CC-GET array was inserted, GET performed and the CC-GET array was removed prior to insertion of the permanent HL14 array for chronic stimulation as a 'bionic ear' hearing prosthesis. CC-GET was undertaken as described for the guinea pig experiments above, but performed bilaterally with randomization of cochlea receiving the pFAR4-CMVp-eGFP DNA and the contralateral cochlea receiving the pFAR4-BDNF-NT3 DNA.

Clinical CIs and speech processors were switched on two weeks after implantation and chronic stimulation in response to environmental sound was presented continuously, except for brief periods when batteries were replaced. Standard clinical frequency allocation tables were used to program speech processors for monopolar stimulation. Biphasic pluses were delivered to each electrode at 500 pulses per second and had a 25 µs phase interval with an 8 µs inter-phase gap. Impedance of the intracochlear electrodes was measured weekly throughout the chronic stimulation period using monopolar stimulation from each intracochlear electrode to the external return electrode via Custom Sound EP 2.0 software (Cochlear ltd.).

Neural response telemetry (NRT) electrocochleography recording of electrically evoked compound action potentials (eCAPS)

The level of the CI electrodes were recorded for both ears in awake animals every 2-3 weeks throughout the chronic stimulation period using Custom Sound EP 2.0 software (Cochlear ltd.). NRT stimulation pulses were delivered to each of the 14 electrodes in monopolar stimulation using monopolar stimulation (MP+1) to the external reference electrode at 25 µs/phase biphasic pulses delivered at 80 pulses per second. ECAPs were recorded at 10 CL (Current Level) intervals from a maximum of 250 CL to below threshold.

Terminal inferior colliculus recordings

Approximately six months after CC-GET and cochlear implantation acute electrophysiological recordings were taken in the inferior colliculus (IC) immediately prior to euthanasia as previously described [5] over a 24-72 hour period. The multi-electrode array IC recording site was driven by each of the CI arrays inserted into control eGFP DNA and neurotrophin DNA – CC-GET treated cochleae. In anesthetized cats 32-channel, 100 µm spacing, recording electrodes on a silicon substrate (NeuroNexus Technologies, USA) were inserted into the IC bilaterally to a depth of ~ 3.5 mm. Anesthesia was induced by intramuscular injection of ketamine (20 mg/kg) and subcutaneous injection of xylazil (2 mg/kg) and maintained by intravenous infusion of sodium pentobarbital (3-8 mg/kg/hr). Spike activity was acquired from the 64 electrodes using a Cerebus acquisition system (Blackrock Microsystems, USA), with sampling of amplified and filtered data at a rate of 30 kHz. Bipolar stimulation delivered to adjacent intracochlear electrodes of the CI array was 100 µs phase duration and 50 µs inter-phase gap, bipolar cathodic-first charge balanced at rate of 4 Hz. This was delivered via an in-house designed multi-channel stimulus generator at CL ranging between 0 and 265 CL (defined by Cochlear Ltd.) where, current in µA = 17.5*(100 ^ (CL/255). Neural activity was detected

based on threshold (4x root-mean-square level) crossings and spikes in a 3 - 45 ms post stimulus window. At each recording site, the spike counts were recorded across 10 trials for each stimulation configuration and current level to calculate spontaneous activity. IC spatial turning curve (STC) width was determined by calculating the average threshold value across the first five electrodes of the 32-electrode IC array for each of the CI electrodes, for each cochlea. The STC width was then defined as the length of the array where electrodes had a threshold value below the average threshold across the first five electrodes.

Plasmid DNA

Plasmids maps for each of the seven DNA molecules used are shown in Figure S1. For transfection, plasmids were purified from *E. coli* using Plasmid Maxiprep Kits (Qiagen) according to the instructions. DNA pellets were resuspended in water for quantification on a Nanodrop Nd-1000 spectrophotometer (ThermoFisher). Quantified DNA was dried using a SpeedVac vacuum concentrator (ThermoFisher) at room temperature and resuspended in isotonic saline or sucrose solutions at the desired concentration for transfection.

Guinea pig tissue collection and analysis

Guinea pigs were euthanized using intraperitoneal injection of pentabarbitol (100 mg/kg). Cochlea were isolated and fixed by perfusion of the cochleae scala with 4% paraformaldehyde in phosphate buffer and submerged in 4% paraformaldehyde at 4 ºC overnight. Perilymph was collected prior to fixation using a 20 µl pipette placed in the round window after perforation of the round window membrane. Perilymph was placed in 1.5 ml microcentrifuge tubes, snap frozen in liquid nitrogen and stored at -80 ºC for BDNF and NT3 ELISA. Samples from brain, heart, blood, mastoid process muscle, liver, kidney, stool and urine were collected and snap frozen in liquid nitrogen for subsequent PCR analysis. After fixation, cochleae were decalcified at 4 ºC in phosphate buffered 8% EDTA (pH7.6) for 2 weeks. Cochleae from experiments using pFAR4-eGFP were hemi sectioned with a scalpel blade and whole-mount imaged on a LSM710 platform (Zeiss) to quantify the number of GFP transfected cells. Cochleae from deafened guinea pig experiments using pFAR4-CMVp-BDNF-NT3 to assess capacity for auditory neuron regeneration were cryoprotected in 30% sucrose prior to cryosection into 50 µm sections, and immunolabelling free-floating for TUBB3 (β3Tubulin; clone TUJ1 Covance) as previously described (Pinyon 2014, Pinyon 2016). Immunolabelled sections were mounted on coverslips using Prolong Gold mounting media (P36931; ThermoFisher) and imaged on a LSM710 platform with a 488 nm argon ion laser. Images were analyzed for neurite regeneration via fluorescence intensity of the TUBB3-labelled auditory neuron peripheral processes using ImageJ (US NIH). The segmented line tool was used to delineate the distal 50% of the osseus spiral lamina region (corresponding to the most pronounced region of neurite density loss at one month – post deafening. The average pixel intensity in this region was determined for sections from each of the four left (deafened with pFAR4-CMVp-BDNF-NT3 delivery) and the control right (deafened only) cochleae.

Cat tissue collection and analysis

Following electrophysiological recordings in the IC, animals were euthanized via intraperitoneal injection of 100 mg/kg pentobarbital and perfused systemically with heparinized 0.9% saline prior to 4% paraformaldehyde at 4 ºC. Cochleae were isolated and fixed for an additional hour submerged in 4% paraformaldehyde at 4 ºC. Cochleae were decalcified in 10% EDTA for 2 weeks then cryoprotected with 30% sucrose before freezing and cryosectioning at 50 µm. Free-floating sections were immunolabelled for TUBB3 (β3Tubulin; clone TUJ1

Covance) and NF200 (neurofilament protein 200 kDa; Cat# N4142, Merck) with respective secondary antibodies goat anti-mouse Alexa Fluor^{TM} 488 (A-11001; ThermoFisher) and goat anti-rabbit Alexa Fluor^{TM} 594 (A-11072; ThermoFisher). All antibodies were used at 1:1000 dilution. Sections were immunolabelled, mounted, imaged and analyzed for neurite regeneration as described above for the guinea pigs, with all sections containing the basal turn osseous spiral lamina (OSL) analyzed (n = between 7 and 10 sections per cochlea, with OSL areas measured between 32,334 and 57,259 square microns. Additionally, average fluorescence intensity of the auditory neuron somata was analyzed in ImageJ (US NIH) using the elliptical selections tool to outline each soma. Four sections from each cochlea were analyzed with between 9 and 61 somata measured from each section. Fluorescence intensity of both cell somata and neurites was measured in the 488 nm argon ion laser channel (TUBB3 immunodetection). At the time of euthanasia additional samples (blood, brain, heart, liver, kidney, lung, spleen, skin, intestine, and muscle from beside the ear canal) were collected and snap frozen in liquid nitrogen for PCR analysis. Samples then underwent DNA extraction and PCR analysis.

Montecarlo analysis of CC-GET electric field thresholds

A finite element model was developed to simulate the electric potential within the media surrounding the 8-electrode cochlea array in the *in vitro* HEK293 cell CC-GET model. Model input parameters include the media volume, the height of the media and the meniscus forming over the array (100 µm), the long semi-axis of the ellipse defining the meniscus, the supply voltage (120 V), and the voltage loss across the electrode-electrolyte bilayer, and the conductivity of the media. Experimentally indeterminate parameters were defined using a Monte-Carlo approach and were randomly selected from a uniform distribution to generate a population of models (n = 10), long-axis of the ellipse defining the meniscus (range 0.3-1.3 mm), voltage loss across the bilayer were random (range 2 – 30 V). This produced a media volume of 21-30 µl, approximating that used experimentally. For each model, the media specific conductivity was estimated by a parameter optimization routine that minimized the difference between the experimentally recorded total system resistance (4000 Ohm) and the model predicted total resistance. For each model (n = 10), the electric field threshold was then estimated by identifying a uniform perimeter symmetrically around the array that enclosed an area equal in size to the area of HEK293 cell transfection measured experimentally.

Measurement of recombinant BDNF and NT3 by ELISA

BDNF and NT3 levels in HEK293 cell culture supernatant (four days post CC-GET) and in guinea pig perilymph samples (2 weeks post CC-GET) were analyzed. ELISA-based quantification of BDNF (R&D Systems catalogue number DY248) and NT3 (R&D Systems catalogue number DY267) was performed as specified by the manufacturer, followed by colorimetric readout measuring absorbance at 415 nm in a FLUOstar microplate reader (PerkinElmer). BDNF and NT3 concentration was extrapolated against a serial dilution of the relevant purified neurotrophin standard.

PCR probe for tissue dispersion of neurotrophin miniplasmid

Samples were thawed and DNA was purified via the HotShot alkaline lysis method [6]. PCR was then performed on the extracted DNA to detect any possible undegraded plasmid DNA still present, using a forward primer located in the codon optimized BDNF coding sequence (5'-GGTTCATCAGGATCGACACTTCC-3') and reverse primer located in the IRES (5'-GGAATGCTCGTCAAGAAGACAGG-3'). PCR was conducted using Taq Polymerase with Thermopol Buffer (New England Biolabs) at 97 °C for 2 min, followed by 40 cycles of 97 °C for 30 s, 64 °C for 30 s and 72 °C for 1 min with a final elongation of 72 °C for 5 min.

Supporting Information – Figures and Tables

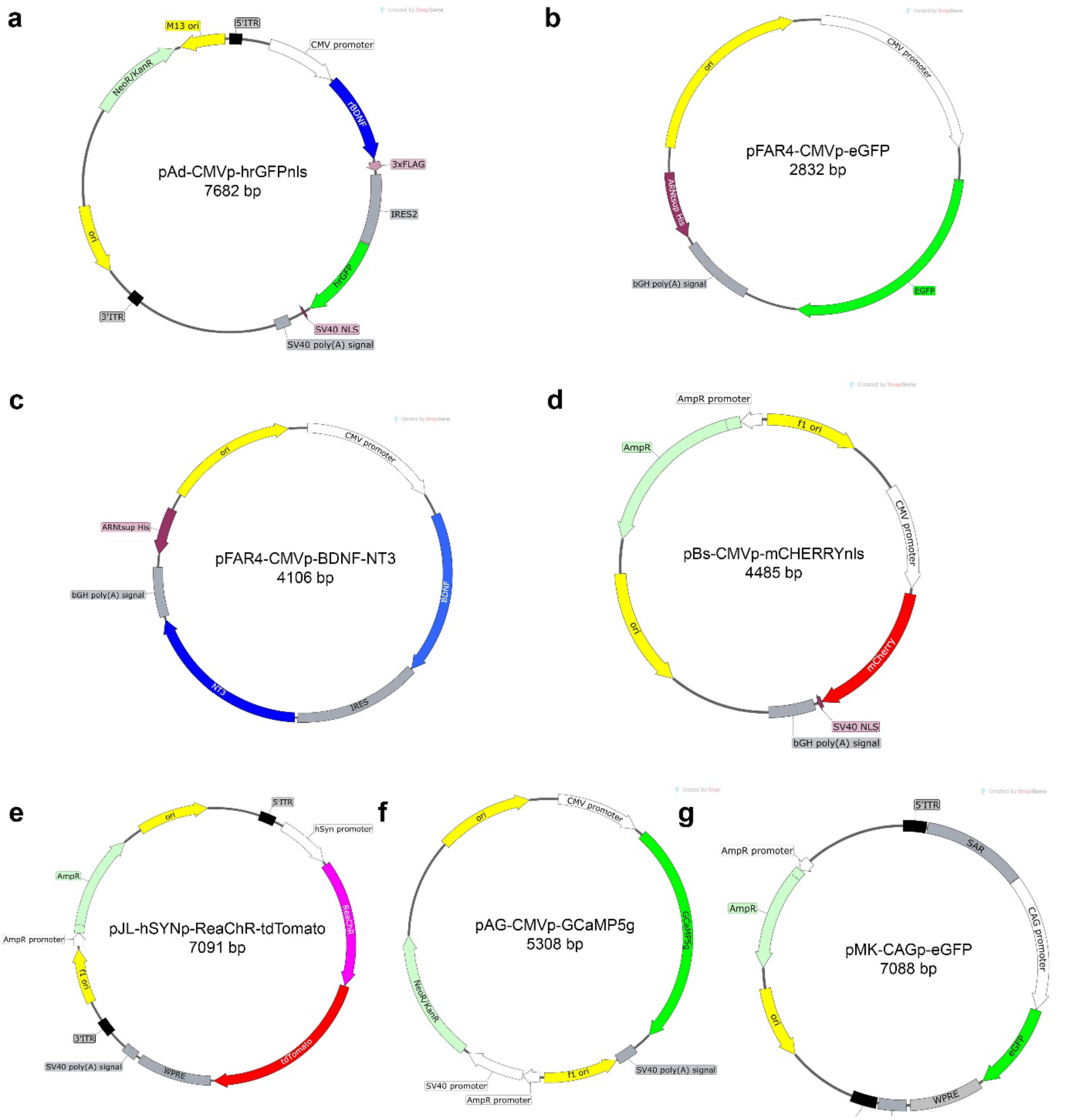

Figure S1. Plasmid maps showing the design of neurotrophin, reporter, and optogenetic expression vectors. (**A**) pAd-CMVp-hrGFPnls, used as a reporter plasmid, containing the nuclear localized humanized renilla GFP (hrGFPnls) reporter coding sequence as the second cistron downstream of an internal ribosome entry sequence (IRES) and the rat brain derived neurotrophic factor (rBDNF) coding sequence driven by the cytomegalovirus promoter (CMVp). (**B**) pFAR4-CMVp-eGFP, a miniplasmid free of antibiotic resistance genes (pFAR) containing the enhanced GFP (eGFP) coding sequence. The pFAR4 plasmid vector enables production of plasmids lacking the conventional antibiotic resistance selection marker genes, pFAR4 plasmids are produced using a genetically modified *E. coli* strain that contains a nonsense mutation in the essential thymidylate synthase (*thyA*) gene generating its thymidine

auxotrophy. pFAR4 plasmids encode a nonsense suppressor t-RNA that restores the ThyA open reading frame and bacterial growth, thus avoiding the use of antibiotic resistance marker for selecting plasmid-containing bacteria. These plasmids are compatible with clinical translation under EMA guidelines for gene electrotransfer, as they lack antibiotic resistance genes, thereby preventing inadvertent conferral of antibiotic resistance in commensal bacterial in the surgical field (after [7]). (**C**) pFAR4-CMVp-BDNF-NT3 contains the same miniplasmid backbone as (B) but with human BDNF and neurotrophin-3 (NT3) cistrons separated by an IRES. (**D**) pBs-CMVp-mCHERRYnls depicts the reporter plasmid used for the experiments shown in Fig. 4. (**E**) pJL-hSYNp-ReaChR-tdTomato contains a fusion protein of the red-shifter channelrhodopsin (ReaChR) and 2x tdTomato reporter under the control of the human synapsin promoter (hSYNp). (**F**) pAG-CMVp-GCaMP5g depicts the genetically encoded Ca^{2+} reporter. (**G**) pMK-CAGp-eGFP contains the eGFP reporter downstream of the cytomegalovirus-Chicken beta-actin-rabbit beta-globin hybrid synthetic promoter (CAGp).

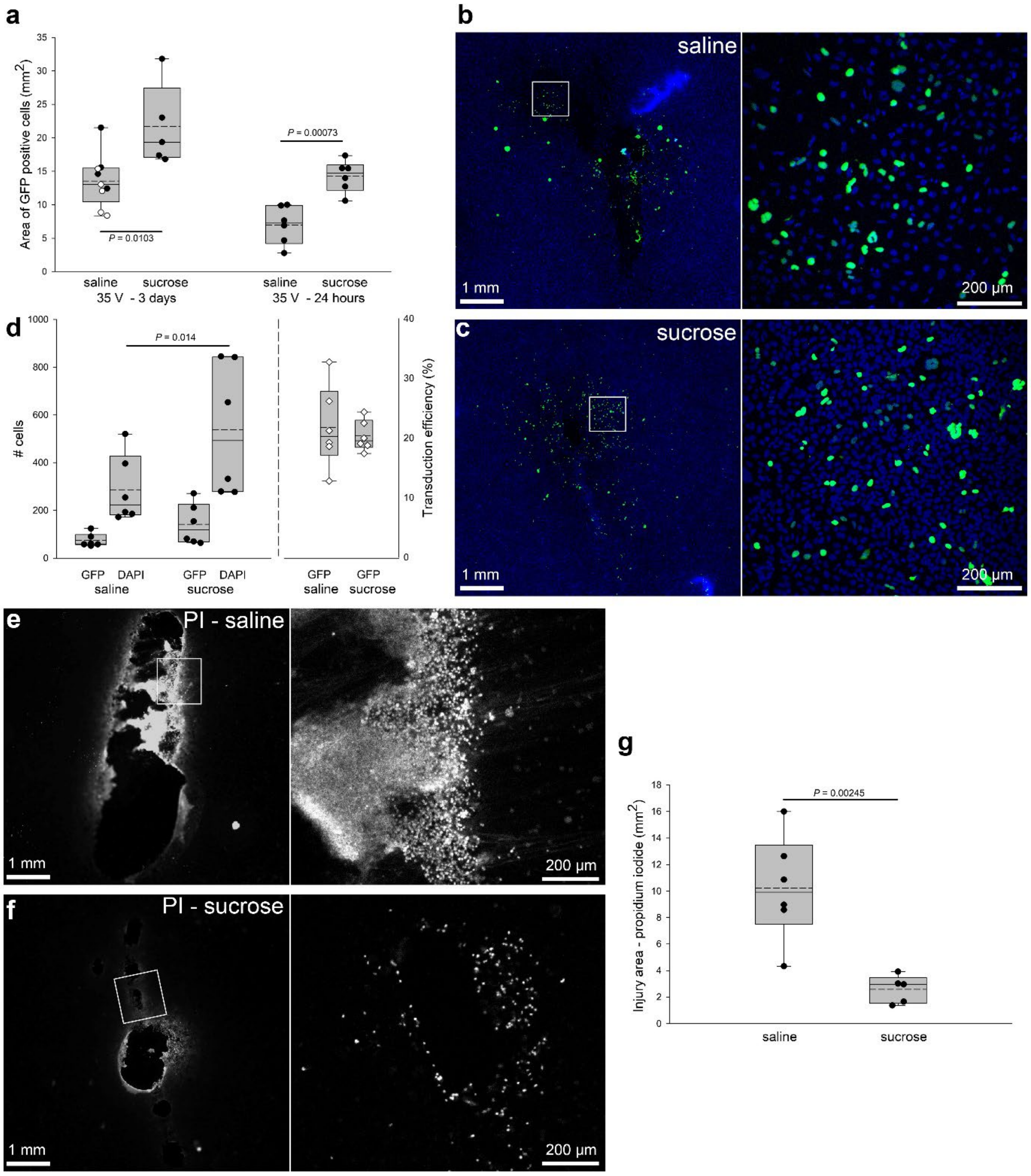

Figure S2. Increase in area of transfected HEK293 cells expressing GFP following CC-GET at equivalent applied voltage using sucrose DNA carrier solution (conductivity-clamping) compared with saline carrier; efficiency and cell injury prevention. (A) 3 day cell culture study (left box plot set): 3 x 100 ms pulses at 35 V applied (V_a) to the gene electrotransfer (GET) probe. pAd-CMVp-hrGFPnls plasmid (2 µg/µl) in 10% sucrose (black circles), or in normal saline (0.9% NaCl) with (black circles) or saline + Tris Buffer (50 mM, pH7.4; open circles; data for saline experiments were pooled; $P > 0.05$, t-test). The size of fields of the nuclear localized GFP+ve cells within HEK293 cell monolayers after 3 days in culture were determined by drawing boundaries around the perimeter of reconstructed montages of confocal images. Box plots show 25% and 75% boundary layers with 95% limits, lines show

median values, dashed lines are means; data from individual experiments overlaid. Student's t-test. Demonstrating the reduction in charge transfer achieved by CC-GET, only 5 mA was required to achieve 35 V_a using the sucrose carrier, compared with 50 mA current pulses for the same V_a when using saline carrier. Data reflected in US patent ref. [8] Right-side box plots report on a 24 hour cell culture study using the same GET parameters for sucrose vs. saline carriers. This replicated the finding of the 3 day cell culture study where sucrose carrier similarly achieved a larger area of transduced cells. This experiment included DAPI staining post-fixation to enable cell counts of regions of interest (ROI) (**B**,**C**; whole field (Olympus Slideview VS200) images showing ROI, detailed in adjacent confocal LSM images, for saline and sucrose carriers respectively). (**D**) These 24 hour cell culture data indicated an equivalent relative gene expression efficiency using saline and sucrose carriers, against a background of lower cell counts in the saline carrier condition (two way ANOVA with Holm-Sidak post-hoc comparison). (**E**,**F**,**G**) demonstrate the impact of the saline carrier for GET on cell viability using the same GET conditions, with propidium iodide (2 µg/ml) added to the culture medium 1 hour after GET to label cells with irreversible electroporation / injury, followed by PFA fixation and imaging. E and F show whole field and detail confocal LSM images of the region in the vicinity of the gene delivery array placement on the HEK293 cell monolayer in saline and sucrose carrier conditions. (G) PI fluorescence delineating cell injury margin surrounding a mechanical artefact associated with contact of the electrodes with the cell layer (as delineated by the sucrose carrier area measurements, where PI signal is minimal) (t-test).

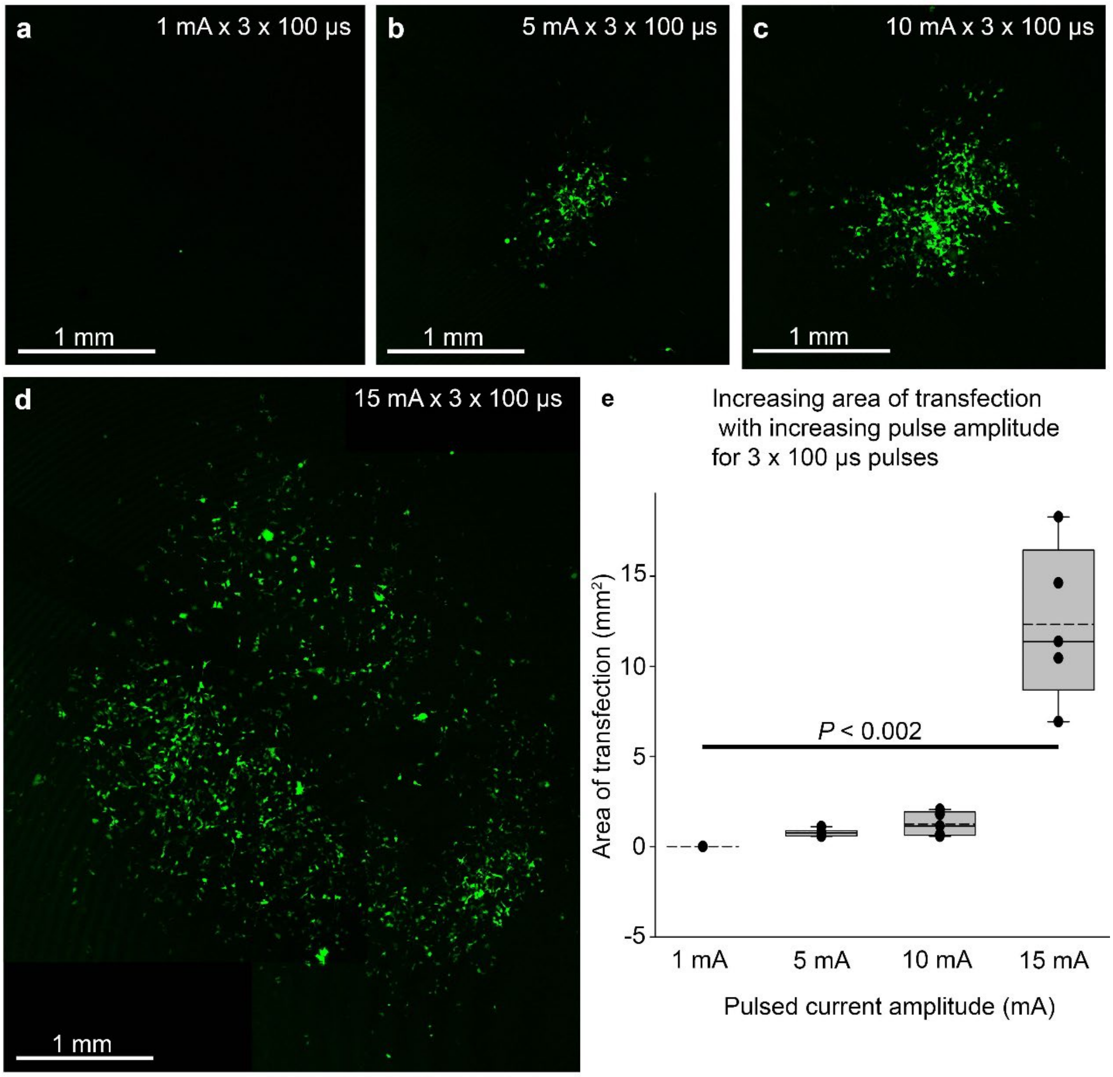

Figure S3. Determination for threshold for reporter plasmid expression with CC-GET DNA electrotransfer using low conductivity-clamping via application of 10% sucrose DNA carrier solution. This CC-GET utilized an adapted 5 mm 8 electrode array (Cochlear Ltd.). (**A-D**) Adherent HEK293 cells on coverslips in culture (3 days) demonstrate GFP expression following electrotransfer of reporter plasmid DNA (pAd-CMVp-hrGFPnls plasmid (2 µg/µl)) using 3 x 100 µs square wave current pulses varying between 1 – 15 mA. (**E**) Box plots show 25% and 75% boundaries, with 95% levels as bars, individual data overlaid (n = 5 per group); lines indicate median, dashed lines indicate means. *P* value shown reflects ANOVA on ranks comparing 15 mA with lower current levels. 1 mA produced minimal expression ($P < 0.001$ vs. 5 mA; t-test). Images from US patent ref. [8]

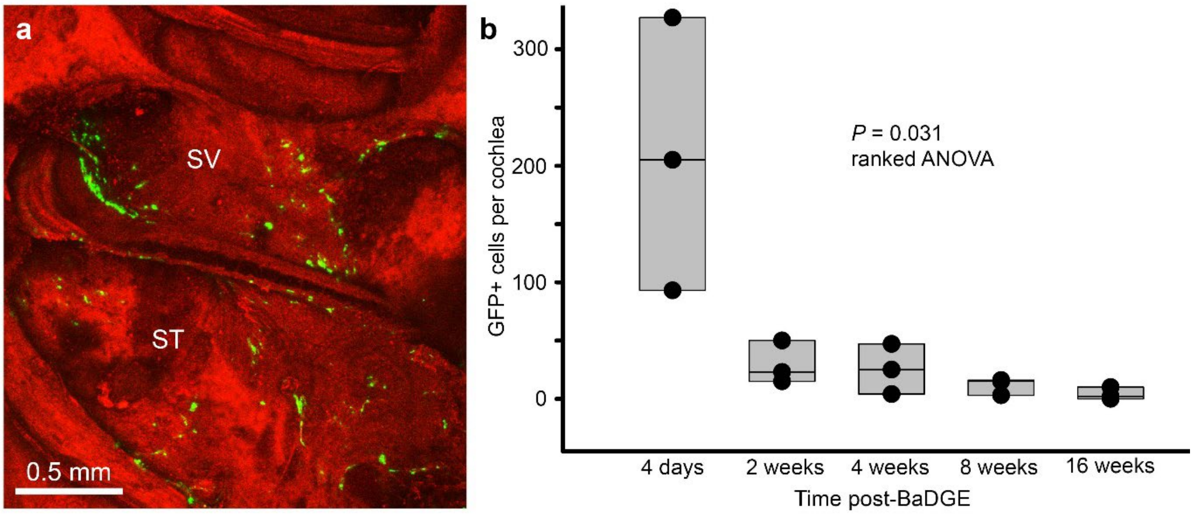

Figure S4. Longitudinal study of CC-GET - directed expression of the pFAR4-CMVp-eGFP reporter plasmid to the cells lining the guinea pig cochlea basal region perilymphatic scalae. The cell counts of GFP+ve mesenchymal cells (green) is highest 4 days after gene electrotransfer and under-represents the total due to clustering. Data from 4 days to 16 weeks after *in vivo* delivery to the basal scala tympani chamber demonstrated significant attenuation in expression. pFAR4-CMVp-eGFP miniplasmid delivered using the clinical CC-GET device (10 x 100 µs x 50 mA pulses). (**A**) reconstructed confocal image stack of the scalae of the basal region of the cochlea achieved by removal of the lateral wall. ST, scala tympani; SV scala vestibuli. Red is autofluorescence. (**B**) Box plots of the counts of GFP expressing cells at time points out to four months following CC-GET delivery of the naked GFP-encoding DNA (25% and 75% boundaries, thin line reflects the median; individual data overlay; ANOVA P value reflects time-dependent decline in expression attributable to turn-over of the targeted mesenchymal cells [3]; n = 15).

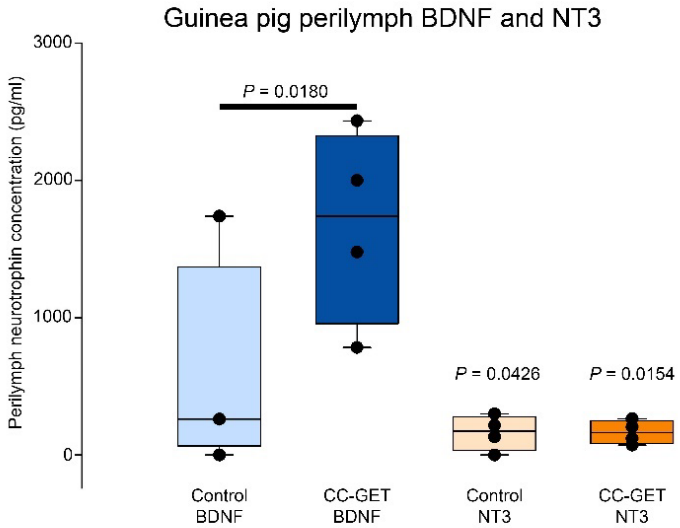

Figure S5. Cochlear perilymph recombinant neurotrophin levels in deafened guinea pigs two weeks after CC-GET delivery of clinical pFAR4-CMVp-BDNF-NT3 DNA. BDNF and NT3 ELISA assays (see Supporting Information - methods). Box plots reflect 25% - 75% boundaries with 95% limits. Median = solid line; mean = dashed line, data overlaid; paired Student's t-test analysis for BDNF comparison against the untreated contralateral (control) cochleae; one-tailed single sample t-tests for NT3 data (n = 4).

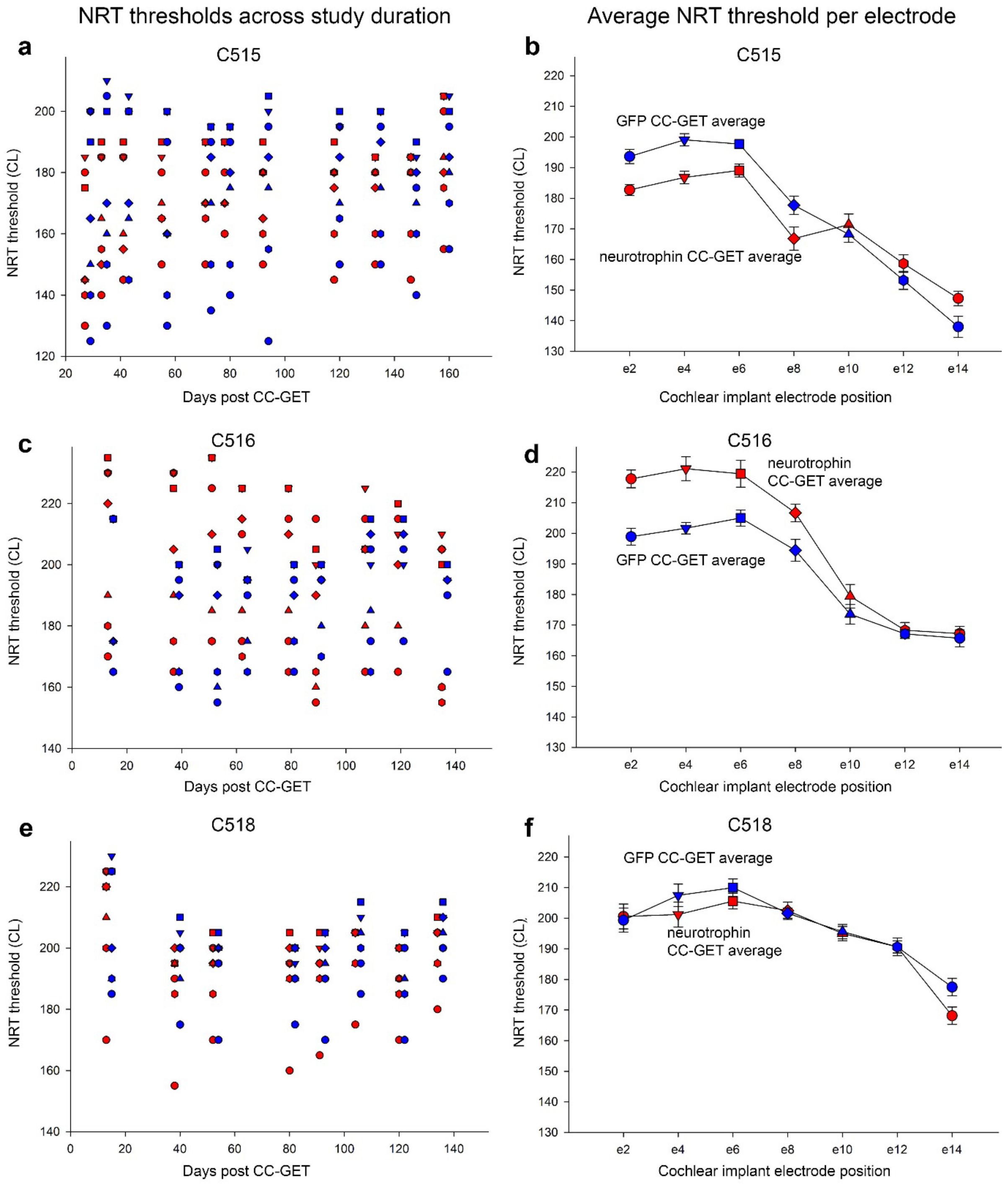

Figure S6. Electrically evoked compound action potential (ECAP) thresholds after CC-GET delivery of neurotrophin or control (eGFP) DNA to the cochleae of deafened cats, supporting long-term hearing using bilateral cochlear implants. (A,C,E), Electrically -evoked compound action potential thresholds measured via cochlear implant nerve response telemetry with monopolar stimulation (MP+1) over time (cat ID: C515, 159 days; C516, 136 days; C518, 135 days) at electrodes e2, e4, e6, e8, e10, e12 & e14 (shown as different symbols

in each vertical stack) for CC-GET delivery of neurotrophin encoding DNA (red; pFAR4-CMVp-BDNF-NT3), and the control DNA (blue; pFAR4-CMVp-eGFP). These data show that the neural recruitment thresholds along the length of the cochlear implant array were stable over time. This is also evident from the limited variance (s.e.m.) around the mean for each electrode across the study period (**B,D,F**). Note the symbols for each electrode in these Figure elements corresponds to the data for individual electrodes at each test day shown in (A,C,E). There was no significant difference between thresholds at different positions along the cochlear implant arrays attributable to a neurotrophin DNA CC-GET effect (P = 0.755), while a pronounced position-dependent effect was evident (P < 0.001; two-way ANOVA), where thresholds declined towards the apex (electrode e14) of the cochlear implant array.

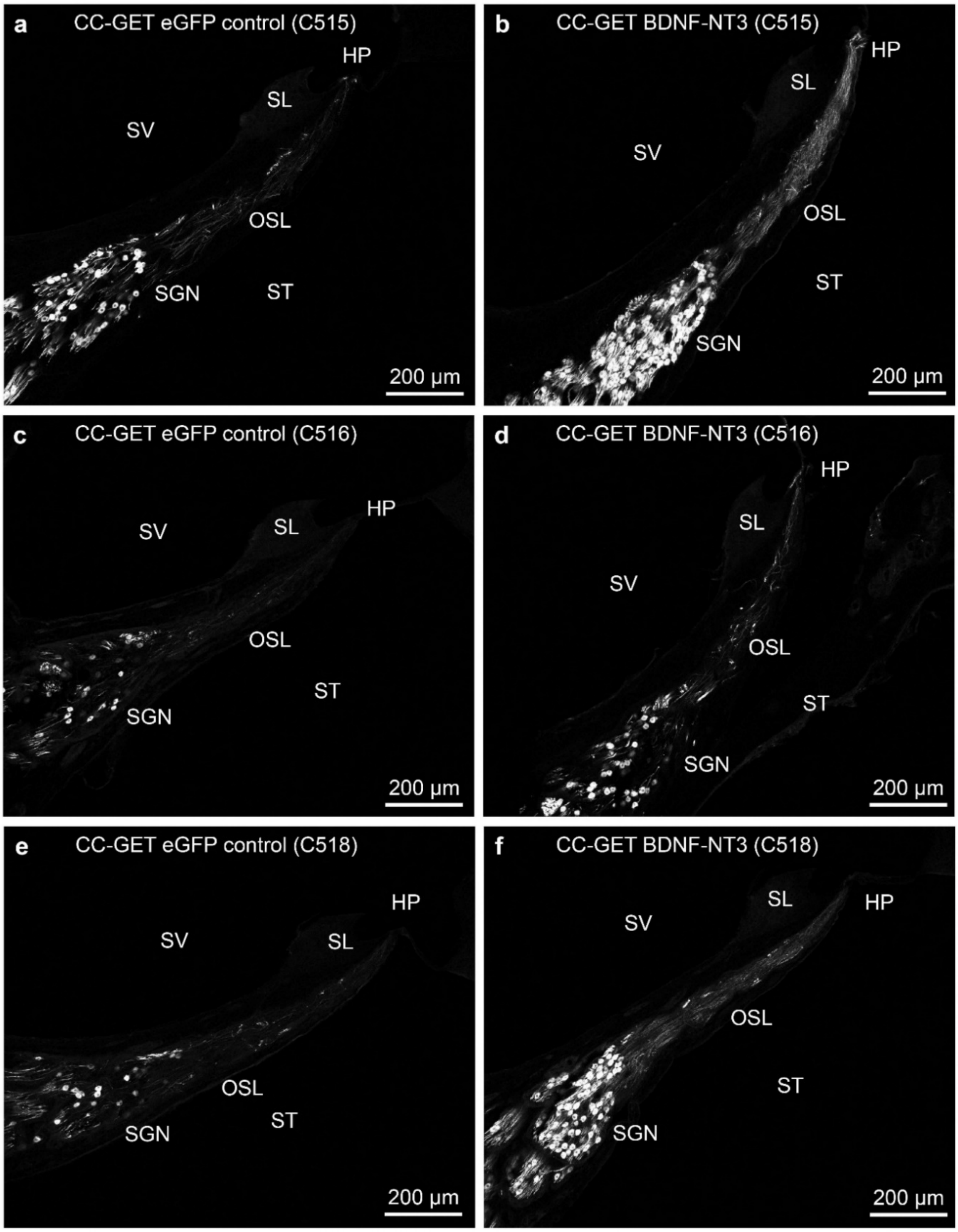

Figure S7. Sustained regeneration of the peripheral spiral ganglion neurites in the cochleae of deafened cats supported by chronic cochlear implant stimulation following CC-GET delivery of neurotrophin-encoding DNA. CC-GET delivery of pFAR4-CMVp-BDNF-NT3 DNA (BDNF-NT3) followed by continuous use of a cochlear implant device sustained the regenerated auditory nerve fibers (135 – 159 days post CC-GET). The controls are the opposite cochleae where CC-GET delivered the pFAR4-CMVp-eGFP - encoding DNA (eGFP). TUBB3 (β III tubulin) immunofluorescence demonstrates enhanced neurite labelling in the osseous spiral lamina (OSL) and in the somata of the spiral ganglion neurons (SGN). HP, habenula perforata; SL, spiral limbus; ST, scala tympani; SV, scala vestibuli.

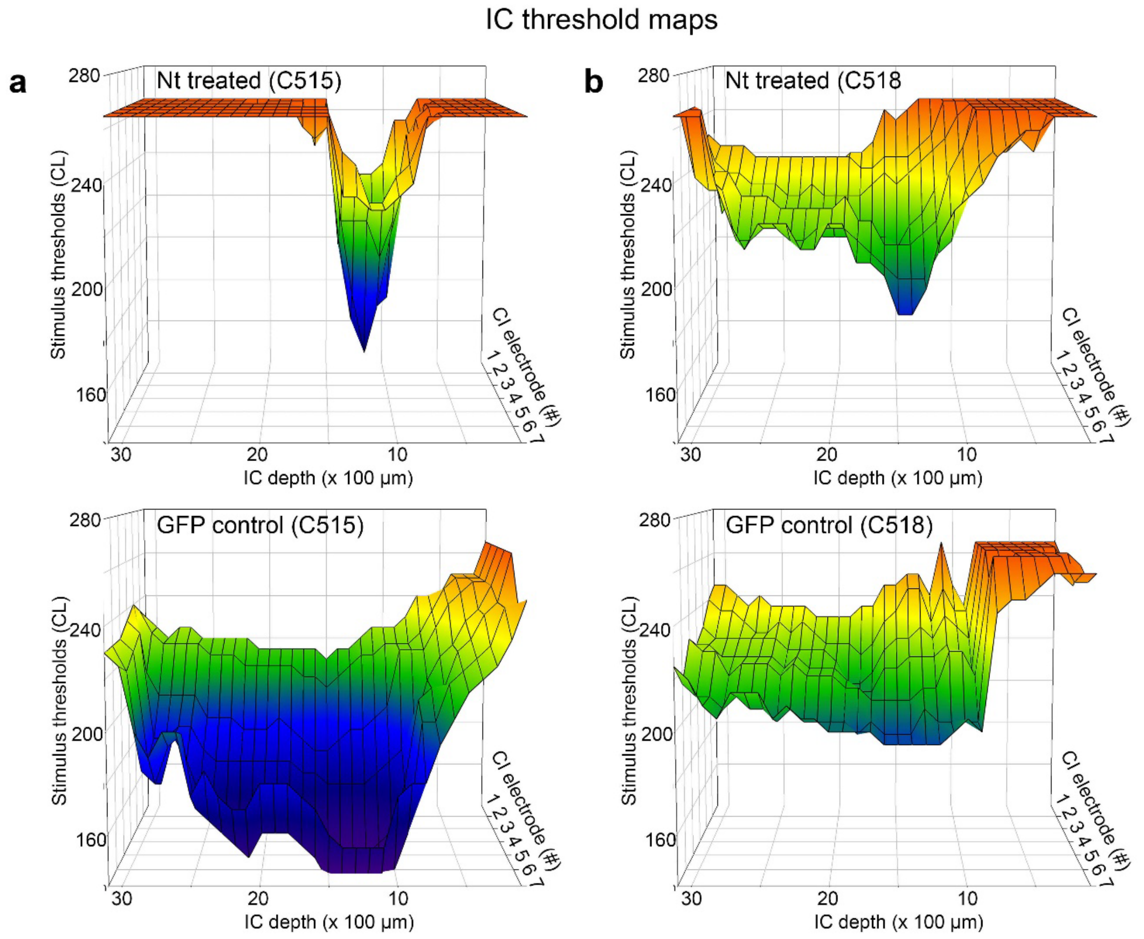

Figure S8. Sharpening of inferior colliculus spatial tuning curves with cochlear CC-GET delivery of BDNF–NT3 neurotrophin DNA followed by chronic cochlear implant stimulation in the deafened cat. Data are recorded from the same 32 channel array inserted into the inferior colliculus, with stimulation across seven cochlear implant electrodes for (**A**) each of pFAR4 CMVp-BDNF-NT3 CC-GET - treated cochlea and (**B**) the contralateral (control) pFAR4 CMVp-eGFP CC-GET – treated cochlea. Following the bilateral CC-GET with (neurotrophin (Nt) encoding DNA into one cochlea and control (eGFP) DNA into the opposite cochlea, the cats received bilateral cochlear implants (CI), which were switched on two weeks after the surgery and remained active for the duration of the study (C516, 135 days; C518 136 days). Data from the third cat is shown in Figure 6. Stimulus threshold of the evoked field potentials was determined as bipolar CI stimulus level (CL, current level).

Table S1. Pulse parameters and resistivity for *in vivo* guinea pig cochlear DNA electrotransfer via the clinical CC-GET device

**Plasmid**<br>**(DNA 2 µg/µl in 10% sucrose carrier)**	**Electrotransfer mean pulse amplitude (mA)***	**Electrotransfer mean applied voltage (V)**	**Local resistance (kΩ)**	**Animal ID**
normal hearing	(50 mA set)			
pFAR4-CMVp-eGFP	50	70	1.40	#3
pFAR4-CMVp-eGFP	50	70	1.40	#4
pFAR4-CMVp-eGFP	35	120	3.43	#6
pFAR4-CMVp-eGFP	29	120	4.14	#7
pFAR4-CMVp-eGFP	50	106	2.12	#8
pFAR4-CMVp-eGFP	43	118	2.74	#9
pFAR4-CMVp-eGFP	42	116	2.76	#11
pFAR4-CMVp-eGFP	48	116	2.42	#14
pFAR4-CMVp-eGFP	45	114	2.51	#15
pFAR4-CMVp-eGFP	50	92	1.80	#16
pFAR4-CMVp-eGFP	49	114	2.32	#17
pFAR4-CMVp-eGFP	50	85	1.70	#18
Mean ± s.e.m., n	45.1 ± 1.98 mA	103.4 ± 5.51 V	2.40 ± 0.24	n = 12
-deafened-	(50 mA set)			
pFAR4-CMVp-BDNF-NT3	38	120	3.15	#234
pFAR4-CMVp-BDNF-NT3	25	120	4.90	#235
pFAR4-CMVp-BDNF-NT3	37	120	3.30	#236
pFAR4-CMVp-BDNF-NT3	23	120	5.30	#237
Mean ± s.e.m., n	30.8 ± 3.92	120 ± 0.00	4.16 ± 0.55	n = 4
		Average across groups	**2.84 ± 0.29** (n = 16)	

Cochlear perilymph reference (baseline)				
	-	-	0.815	#234
	-	-	0.657	#235
	-	-	0.831	#236
Mean ± s.e.m., n			0.77 ± 0.06 kΩ	n = 3

* gene electrotransfer pulse train 10 x 100 µs at set current level of 50 mA; Current levels below 50 mA reflect rail voltage of current controller (Digitimer DS5 analog-controlled stimulator).

Table S2. Pulse parameters and resistivity for *in vivo* cat cochlear DNA electrotransfer via the clinical CC-GET device

**Plasmid (DNA 2 µg/µl in 10% sucrose carrier)**	**Electrotransfer mean pulse amplitude (mA)**	**Electrotransfer mean applied voltage (V)**	**Local resistance (kΩ)**	**Animal ID**
pFAR4-CMVp-eGFP	38 (50 set)	120	3.16	Cat#1 - left
pFAR4-CMVp-eGFP	38 (50 set)	120	3.16	Cat#1 - right
pFAR4-CMVp-eGFP	23 (40 set)	120	5.22	C515 - left
pFAR4-CMVp-BDNF-NT3	34 (40 set)	120	3.53	C515 - right
pFAR4-CMVp-BDNF-NT3	35 (40 set)	120	3.43	C518 - left
pFAR4-CMVp-eGFP	35 (40 set)	120	3.43	C518 - right
pFAR4-CMVp-BDNF-NT3	34 (40 set)	120	3.53	C516 - left
pFAR4-CMVp-eGFP	36 (40 set)	120	3.33	C516 - right
pFAR4-CMVp-eGFP	40 (40 set)	95	2.38	C517 - left
pFAR4-CMVp-BDNF-NT3	28 (40 set)	120	4.29	C517 - right
	Avg current 34.1 ± 1.6 mA	117.5 ± 2.5 V Average Voltage achieved across groups	3.55 ± 0.24 kΩ (n = 10) Average ± s.e.m resistance across groups	

Table S3. Pulse parameters and brain resistivity for *in vivo* DNA electrotransfer in guinea pig brain using the clinical CC-GET device

Plasmid (DNA 2 µg/µl in 10% sucrose carrier)		mean pulse amplitude (mA)	mean applied voltage (V)	Local resistance (kΩ)	Pulse profile (number x duration)	Animal ID
pMK-CAGp-eGFP		10	40	4	3 x 100 ms	GP457
pAd-CMVp-hrGFP*		10	25	2.5	3 x 100 ms	GP452
pFAR4-CMVp-eGFP		50	90	1.8	10 x 100 µs	GP240
pFAR4-CMVp-eGFP		50	90	1.8	10 x 100 µs	GP239
pJL-hSYNp-ReaChR-tdTomato + pAG-CMVp-GCaMP		50	-	-	10 x 100 µs	GP232
pJL-hSYNp-ReaChR-tdTomato		25	70	2.8	10 x 100 µs	GP231
pJL-hSYNp-ReaChR-tdTomato		25	60	2.4	10 x 100 µs	GP230
pJL-hSYNp-ReaChR-tdTomato		10	35	3.5	10 x 100 µs	GP229
pJL-hSYNp-ReaChR-tdTomato		50	90	1.8	10 x 100 µs	GP228
		**Range**	**Range**	**Average**		
		10-50 mA	25-90 V	2.58 ± 0.29 kΩ		

* = 6.5% glucose

<u>Primary figure commentary (Figures 1, 2, 4, 6 and 7)</u>

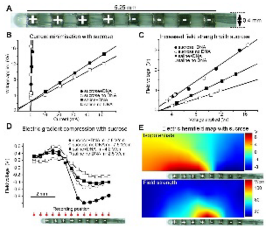

Figure 1. Non-conductive sucrose carrier amplifies bionic array electric field strength.
This figure demonstrates the effect of substituting a standard saline (0.9% NaCl) – based carrier for plasmid DNA GET, with isotonic 10% sucrose. (**A**) 8-electrode GET array depicting four ganged anodes (+) and cathodes (-), where this longitudinal extension of the electrode poles contributes to the electric field 'lensing'. Each of the full ring Pt/Ir electrodes is 350 µm in width, 400 µm diameter, with 350 µm spacing. (**B**) Using a current-controlled stimulator, the voltage applied to the electrodes approaches the maximum driving voltage of the stimulator with minimal current when sucrose is the carrier. Addition of DNA (sheared salmon sperm DNA, average size 2 kb, 2 µg/µl) to the normal saline carrier (0.9% NaCl) reduces conductivity, so that the voltage applied (V_a) to the electrode increases, which benefits GET, however, there is no appreciable additional effect of the DNA within sucrose carrier. (**C**) Measured at a single point relative to the array (e7, 0.5 mm), the rate that the sampled field voltage (V_f) increases with increasing V_a is enhanced for DNA + saline, compared with saline alone (augmenting the advantage for GET at the level V_a shown in (B)); note however, that conductivity clamping with sucrose produces a further augmentation of the V_f, for a given V_a. (**D**) Extending from the point measurement of V_f in (C), here V_f is measured along the length of the array, 0.5 mm lateral. The greatest change in Vf with distance (maximum electric field strength; $\Delta V_f/\Delta d$) occurs across the null point ($V_f = 0$ V) at the transition between the anode and cathode poles. This highlights the point that the absolute voltage (in the field) is irrelevant, and this close-field electric 'lensing' effect provides compression of the electric field not achievable with conventional GET electrode configurations due to current spread. (**E**) The isopotential map (hemifield; upper graphic) represents extended sampling of V_f within the region around the electrode array with sucrose carrier, enabling derivation of the (2D) electric field map (at $V_a = 40$ V, lower graphic), which is circular and equivalent to suprathreshold GET field strengths for several mm orthogonal to the array.

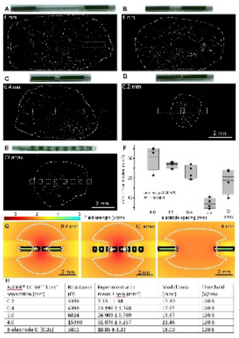

Figure 2. Electric field 'lensing' via the CC-GET array enables spatial focusing of gene expression.
Control of gene expression with fixed charge transfer using electric field 'lensing', via change in separation between linear extended electrode elements was used to model GET electric field threshold. Montecarlo modelling established the electric field threshold for DNA electrotransfer, utilizing data from GFP reporter expression (threshold biomarker) in HEK293 cell monolayers. The effect of separation of the CC-GET 'electro-lens' was evaluated using a modified GET array where the four ganged electrodes for one pole of the multi-electrode array were substituted for a single longitudinally extended Pt/Ir electrode of 2 mm length x 400 µm diameter, with variation of the separation with the other electrode surface, on a silicone backbone (shown in insets). CC-GET was performed using a GFP reporter plasmid including a nuclear localization signal to discretely resolve HEK293 cells expressing the transgene construct. To optimally model 'real world' intracochlear delivery we used a modified 'conductivity clamping' carrier solution comprising 9% sucrose and 0.09% saline (pH 7.4, 0.5 mM NaOH), with 2 µg/µl pAd-CMVp-hrGFPnls plasmid DNA (~ 12 $\mu S.cm^{-1}$ conductivity). The size of the transfected field of HEK293 cells was assessed using a fixed suprathreshold pulse train (10 x 100 µs, 400 µs interpulse interval, 30 mA square wave pulses; requiring 120 V applied; ~ 30 µC charge transfer; ~ 4 kΩ local resistance). For this fixed charge transfer, the area of transfected GFP+ve cells was found to be scalable by a factor of 4.4, from 7.13 ± 1.34 mm^2 to 31.08 ± 3.24 mm^2, when the anode - cathode separation was varied between 0.2 mm and 4 mm respectively (**A-D, F**). ANOVA indicated the significant effect on gene expression of increasing the separation between the electrodes ($P = 0.001$).The use of two elongated electrode surfaces was found to accurately model the cochlear implant-style multi-electrode array, where the 0.4 mm separation resulted in an average area of GFP expressing cells of 23.20 ± 3.50 mm^2 ; not significantly different from that achieved using the reference 8 electrode GET array with equivalent separation between the gangs of four anodes and four cathodes (18.85 ± 3.33 mm^2, $P = 0.500$ ANOVA, **E, F**). (**F**) Boxplots of areas of transfected cells relative to electrode separation show 25% and 75% boundaries, with 95% limits as bars; lines indicate median, dashed lines indicate means, data overlaid (n = 4 per group). (**G**) Montecarlo modelling of electric fields across the configurations (*see Supporting Information – Materials and Methods*). The dashed perimeter, established by comparison with the perimeter of the field of transfected HEK293 cells reflects the electric field threshold for DNA electrotransfer ($\Delta V_f/\Delta d$ = 120.8 ± 20.2 V/cm; mean ± SD.). (**H**) The predicted electric field threshold (120.8 V/cm) was validated *in silico* by further simulations using the clinical CC-GET array. The long-axis of the fluid ellipse around the electrodes defining the meniscus and the voltage loss across the bilayer were set as the mean of the n = 10 random values generated in (G), 0.861 mm and 14.6 V respectively. Volume of solution was 26 µl. The media electrical conductivity was taken as the average of values estimated in (G), 0.437 S/m. Supply voltage was 120 V. The data summarizes the validation simulations that accurately predicted the surface area of the field of cells with GFP reporter expression (suprathreshold GET electric field strength) using the different 'lens' separations (spacing between the opposing conductive surfaces). Images (A-D) modified from Patent ref. [9]

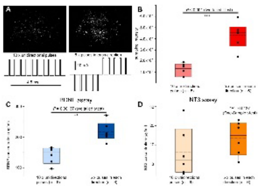

Figure 4. Validation of recombinant BDNF and NT3 protein synthesis in HEK293 cells using the clinical CC-GET device to deliver pFAR4-CMVp-BDNF-NT3 miniplasmid and pBs-CMVp-mCHERRYnls reporter DNA. The plasmid DNAs were delivered as a 30 µl cocktail (1 µg/µl each) in 9% sucrose + 0.09% NaCl + 0.5 mM NaOH carrier, and CC-GET performed using an 8 node electrotransfer array in tandem configuration. (**A** and **B**) Either 10 x 100 µs x 20 mA (400 µs interpulse interval) positive pulses, or 5 positive then 5 negative pulses, were utilized for CC-GET. Switching of electrode polarity within the pulse train provided a significant increase in mCherry reporter fluorescence, as well as increases in BDNF, (**C**) and NT3 (**D**) synthesis. This improved expression achieved by changing polarity may be attributable to the bias of DNA electrotransfer to the cathode-facing cell surface saturating binding efficiency. Overall, the use of alternating monophasic pulses achieved significant improvement of both BDNF and NT3 production (P = 0.001; ANOVA). ELISA analysis methodology is described in Supporting Information – Materials and Methods. Data reflected in Figure 12 Patent ref.. [9] Cells were imaged and supernatant collected at 4 days post CC-GET.

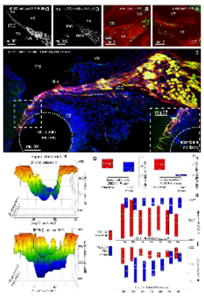

Figure 6. Clinical CC-GET neurotrophin gene augmentation with chronic cochlear implant stimulation achieves long-term rescue of regenerated spiral ganglion neurites and reset of central auditory tuning in the cat inferior colliculus.

(**A** and **B**) Localization of pFAR4-CMVp-eGFP expression in the lower turn of scala tympani (ST) is matched to the prior modelling of the clinical CC-GET device electric field profile (*in vivo* study of normal hearing cat, 72 hours post - CC-GET). Low magnification complementary images of hemi-sectioned cochlea (A) lateral aspect facing spiral ligament of otic capsule, (B) central modiolar wall show clusters of GFP reporter fluorescence positive mesenchymal cells in a cochlear preparation following decalcification (background autofluorescence in the red channel resolves structure). Note the absence of the GFP signal in the more apical ST region in (B), indicating the dependency of expression on the focused electric field generated by the CC-GET device. When this plasmid DNA is replaced with the therapeutic neurotrophin-encoding DNA (pFAR4-CMVp-BNDF-NT3), these cells secrete BDNF and NT3, establishing a guidance cue for directed regrowth of the spiral ganglion neurites. (**C** to **E**) regeneration of the spiral ganglion peripheral neurites is retained at the six-month endpoint in deafened cats. The majority of the regenerated fibers extend out though the osseous spiral lamina (OSL) to the normal insertion point to the sensory epithelium (habenula perforate, HP), and continue laterally beyond the insertion point of the basilar membrane with the spiral ligament, with progressive dissipation of the fiber fascicle as individual branches project through the mesenchymal cell layer in close juxtaposition to the cochlear implant electrode array. The cochlear implant array was inserted immediately after withdrawal of the clinical CC-GET device, and actively stimulated the spiral ganglion neurons to support hearing for 8 hours per day for the duration of the study. (C) control cochlea (pFAR4-CMVp-eGFP DNA delivery) TUBB3 (β3-Tubulin) immunolabelling shows scarcity of auditory nerve fibers in the osseous spiral lamina compared with the pFAR4-CMVp-BNDF-NT3 DNA - treated cochlea from the same animal, shown in (D). (E) double immunolabeling for TUBB3 and neurofilament 200, with DAPI fluorescence for nuclear labelling in a different deafened cat following neurotrophin DNA treatment. RW, round window membrane, the insertion point for the CC-GET device and then the cochlear implant array; SV, scala vestibuli; SM, scala media. Due to the restricted geometry of ST above the basal region of the cat cochlea, only the eight ganged half-band electrodes in the tip (zone 1) of the clinical CC-GET device were used for electrotransfer (10 x 100 µs x 50 mA pulses delivered at 2 kHz using a constant current controller; compliance limited to an average of 34 mA). Either the control pFAR4-CMVp-eGFP plasmid DNA, or the therapeutic pFAR4-CMVp-BDNF-NT3 plasmid DNA (2 µg/µl in 10% sucrose carrier, with 0.5 mM NaOH) was delivered into the cochlear ST through the tip of the clinical CC-GET device (50 µl at 10 µl / min), immediately prior to the electrotransfer current pulses. (**F**) Somata from cryosections (symbols distinguish average data from the three cats). (**G**) inferior colliculus auditory threshold maps from a deafened cat receiving neurotrophin-encoding DNA via CC-GET into one cochlea and eGFP–encoding DNA into the opposite cochlea, followed by continuous use of bilateral cochlear implant devices. The data reflect threshold auditory- evoked potentials recorded from 32 electrodes spanning the depth of the inferior colliculus (IC) elicited by bipolar stimulation of the respective cochlear implant arrays (electrodes 4 – 10). Stimulation of the cochlear implant array within the neurotrophin gene augmentation – treated cochlea exhibited enhanced spatial tuning at the IC array compared with that driven by stimulation of the opposite (control) cochlea. (**H**) IC spatial tuning curve (STC) width is significantly sharper as a result of the cochlear neurotrophin-gene augmentation treatment. The effect on spatial tuning curve width is most

pronounced with stimulation of cochlear implant electrode positions e6 – e9, which closely corresponds to the peak of the CC-GET electric field. The boxplot indicates 25% - 75% limits and the different symbols reflect the average for each cat (solid black line is the median, white dashed line is the mean). (**I**) Boxplots of average stimulus thresholds across all 32 IC electrodes for bipolar stimulation at each of the 7 cochlear implant electrodes, for three cats, with neurotrophin treatment in one cochlea and eGFP -encoding DNA treatment in the opposite cochlea. The data show that the cochlear neurotrophin gene augmentation treatment results in an overall reduction in excitability, which is associated with sharpening of spatial tuning.

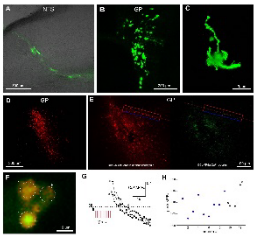

Fig. 7. CC-GET gene delivery to guinea pig brain achieves targeted optogenetic neuromodulation. Enablement of naked plasmid DNA electrotransfer to the brain via close-field conductance-clamping using sucrose carrier. (**A**) GFP-positive neurons in the nucleus of the tractus solitarius (NTS) region of the brainstem identified by confocal fluorescence imaging in a 50 µm cryosection taken 3 days after *in vivo* CC-GET (pFAR4-CMVp-eGFP plasmid; 10 x 100 µs x 50 mA). (**B**) Detail of eGFP-positive globus pallidus (GP) neurons imaged 7 days post CC-GET (pMK-CAGp-eGFP; 3 x 100 ms x 10 mA pulses requiring ~35V applied voltage; 50 µm cryosection). (**C**) Detail of a transfected GP neuron from the same experimental animal providing the data shown in (B). (**D**) A field of dual-plasmid transduced neurons within GP (pAG-CMVp-GCaMP5g + pJL-hSYNp-ReaChR-tdTomato; 10 x 100 µs x 50 mA; tdTomato-tagged ReaChR channel rhodopsin expression shown (300 µm vibratome section imaged via LSM as an image stack at 4 µm z resolution (95 µm total depth, 3D projection). (**E**) Detail of dual expression of ReaChR channel rhodopsin (red channel) and GCaMP5g Ca^{2+} reporter (green channel) from the same animal as experimental data shown in (D). Boxed insets highlight neurons co-expressing ReaChR and GCaMP5g. (**F**) Detail of regions of interest delineating three neurons co-expressing ReaChR and GCaMP5g, where the ReaChR ion channels were photo-activated. (**G**) Intracellular Ca^{2+} responses to 561 nm pulsed laser activation of the three neurons shown in (F) measured via the GCaMP5g green fluorescence emission (488 nm excitation). (**H**) Peak GCaMP5g Ca^{2+} fluorescence responses for 12 neurons with photo-activation of the co-expressed ReaChR channel rhodopsin. Images from (B) and (C) are from patent ref. [8]

References

[1] G. D. Housley, C. J. Browne, E. N. Crawford, M. Klugmann, N. H. Lovell, and J. L. Pinyon, "Cochlear Implant Close-Field Electroporation," in *Handbook of Electroporation*, D. Miklavčič, Ed.: Springer, 2016, pp. 1-20.

[2] C. J. Browne *et al.*, "Mapping of bionic array electric field focusing in plasmid DNA-based gene electrotransfer," *Gene Ther,* vol. 23, no. 4, pp. 369-79, Apr 2016.

[3] J. L. Pinyon, M. Klugmann, N. H. Lovell, and G. D. Housley, "Dual-Plasmid Bionic Array-Directed Gene Electrotransfer in HEK293 Cells and Cochlear Mesenchymal Cells Probes Transgene Expression and Cell Fate," *Hum Gene Ther,* vol. 30, no. 2, pp. 211-224, Feb 2019.

[4] R. K. Shepherd *et al.*, "Platinum dissolution and tissue response following long-term electrical stimulation at high charge densities," *J Neural Eng,* Feb 12 2021.

[5] S. S. George, A. K. Wise, M. N. Shivdasani, R. K. Shepherd, and J. B. Fallon, "Evaluation of focused multipolar stimulation for cochlear implants in acutely deafened cats," *J Neural Eng,* vol. 11, no. 6, p. 065003, Dec 2014.

[6] G. E. Truett, P. Heeger, R. L. Mynatt, A. A. Truett, J. A. Walker, and M. L. Warman, "Preparation of PCR-quality mouse genomic DNA with hot sodium hydroxide and tris (HotSHOT)," *Biotechniques,* vol. 29, no. 1, pp. 52, 54, Jul 2000.

[7] C. Marie, G. Vandermeulen, M. Quiviger, M. Richard, V. Preat, and D. Scherman, "pFARs, plasmids free of antibiotic resistance markers, display high-level transgene expression in muscle, skin and tumour cells," *J Gene Med,* vol. 12, no. 4, pp. 323-32, Apr 2010.

[8] G. D. Housley, N. H. Lovell, J. Pinyon, E. N. Crawford, and C. Browne, "Electroporation system for controlled localized therapeutics delivery. *US Patent No. US 11,213,671 B2*," 2022.

[9] G. D. Housley, J. Pinyon, N. H. Lovell, and A. A. Abed, "Method and system for controlling molecular electrotransfer," *WIPO - PCT WO 2020/118383 A1,* 2020.
